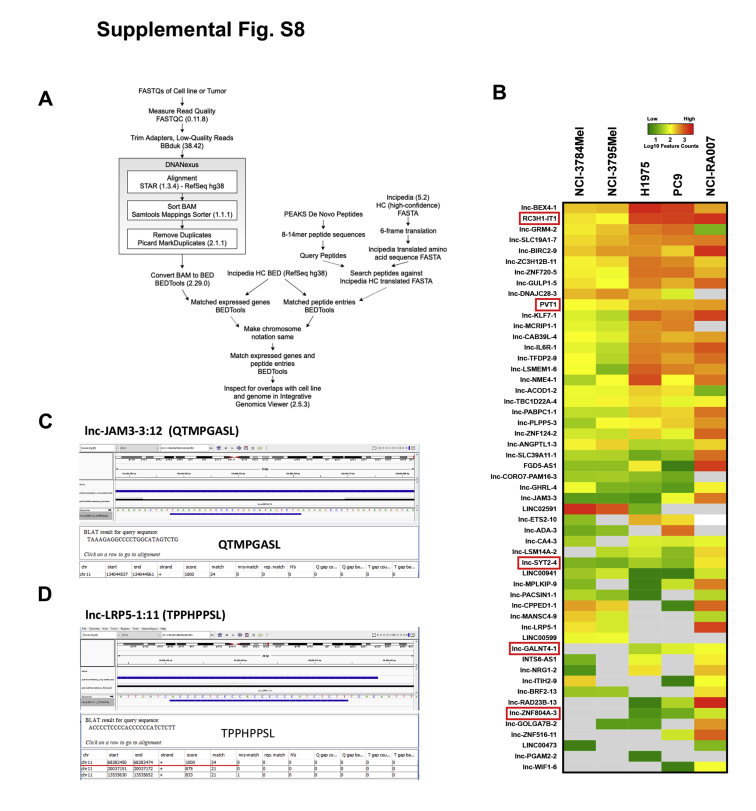# Proteogenomic Analysis Unveils the HLA Class I-Presented Immunopeptidome in Melanoma and EGFR-Mutant Lung Adenocarcinoma

**DOI:** 10.1016/j.mcpro.2021.100136

**Published:** 2021-08-13

**Authors:** Yue A. Qi, Tapan K. Maity, Constance M. Cultraro, Vikram Misra, Xu Zhang, Catherine Ade, Shaojian Gao, David Milewski, Khoa D. Nguyen, Mohammad H. Ebrahimabadi, Ken-ichi Hanada, Javed Khan, Cenk Sahinalp, James C. Yang, Udayan Guha

**Affiliations:** 1 Thoracic and GI Malignancies Branch, Center for Cancer Research, NCI, NIH, Bethesda, Maryland, USA; 2 Surgery Branch, Center for Cancer Research, NCI, NIH, Bethesda, Maryland, USA; 3 Genetics Branch, Center for Cancer Research, NCI, NIH, Bethesda, Maryland, USA; 4 Cancer Data Science Laboratory, Center for Cancer Research, NCI, NIH, Bethesda, Maryland, USA; 5 Department of Computer Science, Indiana University, Bloomington, Indiana, USA; 6 Bristol-Myers Squibb, Lawrenceville, New Jersey, USA

**Keywords:** immunotherapy, HLA immunopeptidome, neoantigen, lung cancer, melanoma, ACN, acetonitrile, ACT, adoptive T-cell therapy, ALC, average local confidence, ATCC, American Type Culture Collection, BLAT, BLAST-like Alignment Tool, CCR, Center for Cancer Research, CG, cancer germ line, CIP2A, cellular inhibitor of PP2A, COSMIC, Catalogue of Somatic Mutations in Cancer, DB, database, DMSO, dimethyl sulfoxide, EGFR, epidermal growth factor receptor, EIF, eukaryotic initiation factor, FBS, fetal bovine serum, FDR, false discovery rate, GWIPS, Genome-Wide Information on Protein Synthesis, HI, hydrophobicity index, HLA, human leukocyte antigen, IEDB, Immune Epitope Database and Analysis Resource, IGV, Integrative Genomics Viewer, INDEL, insertion and deletion, IPA, ingenuity pathway analysis, LDHC, lactate dehydrogenase C, lncRNA, long noncoding RNA, MS/MS, tandem MS, NCI, National Cancer Institute, NGS, next-generation sequencing, PTM, post-translationally modified, pyroGlu, pyroglutamate, RT, retention time, SNV, single nucleotide variant, SSP, sequence-specific primer, TAP, transporter associated with antigen processing, TMB, tumor mutational burden, VCF, variant call format, WES, whole-exome sequencing

## Abstract

Immune checkpoint inhibitors and adoptive lymphocyte transfer–based therapies have shown great therapeutic potential in cancers with high tumor mutational burden (TMB), such as melanoma, but not in cancers with low TMB, such as mutant epidermal growth factor receptor (EGFR)–driven lung adenocarcinoma. Precision immunotherapy is an unmet need for most cancers, particularly for cancers that respond inadequately to immune checkpoint inhibitors. Here, we employed large-scale MS-based proteogenomic profiling to identify potential immunogenic human leukocyte antigen (HLA) class I-presented peptides in melanoma and EGFR-mutant lung adenocarcinoma. Similar numbers of peptides were identified from both tumor types. Cell line and patient-specific databases (DBs) were constructed using variants identified from whole-exome sequencing. A *de novo* search algorithm was used to interrogate the HLA class I immunopeptidome MS data. We identified 12 variant peptides and several classes of tumor-associated antigen-derived peptides. We constructed a cancer germ line (CG) antigen DB with 285 antigens. This allowed us to identify 40 class I-presented CG antigen–derived peptides. The class I immunopeptidome comprised more than 1000 post-translationally modified (PTM) peptides representing 58 different PTMs, underscoring the critical role PTMs may play in HLA binding. Finally, leveraging *de novo* search algorithm and an annotated long noncoding RNA (lncRNA) DB, we developed a novel lncRNA-encoded peptide discovery pipeline to identify 44 lncRNA-derived peptides that are presented by class I. We validated tandem MS spectra of select variant, CG antigen, and lncRNA-derived peptides using synthetic peptides and performed HLA class I-binding assays to demonstrate binding to class I proteins. In summary, we provide direct evidence of HLA class I presentation of a large number of variant and tumor-associated peptides in both low and high TMB cancer. These results can potentially be useful for precision immunotherapies, such as vaccine or adoptive cell therapies in melanoma and EGFR-mutant lung cancers.

## Highlights

• Proteogenomics identified ∼35,000 class I-presented peptides.
• CG antigen and PTM peptides identified in melanoma and lung cancer.
• *De novo* search identified variant and lncRNA-derived peptides.
• A new strategy to identify class I-presented lncRNA-derived peptides developed.

## Graphical Abstract

**Figure:**
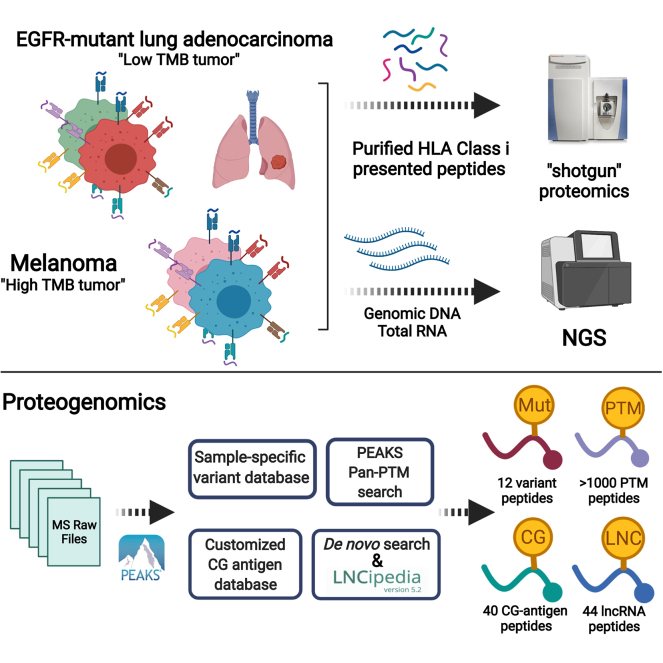

## In Brief

Cancer immunotherapy is ineffective in low TMB EGFR-mutant lung adenocarcinoma. Qi *et al.* performed a comprehensive proteogenomic profiling of HLA class I-presented immunopeptides in high TMB melanoma and low TMB EGFR-mutant lung cancer. Similar numbers of immunopeptides were identified from both. Variant, CG antigen, PTM, and lncRNA-derived peptides were identified. A novel strategy to identify lncRNA-derived peptides was developed. The direct identification of class I-presented immunopeptides will potentially accelerate precision immunotherapy for low TMB tumors.

Cancer immunotherapy has become an essential component of therapy for diverse cancers. The treatment outcome and patient survival rate are positively correlated with their tumor mutational burden (TMB) (1, 2, 3). Epidermal growth factor receptor (EGFR)-mutant lung adenocarcinoma occurs predominantly in never smokers or oligosmokers and exhibits a relatively low TMB (4). Immunotherapy has been less successful in EGFR-mutant lung cancer, in part, because of its low TMB (5, 6). In contrast, melanoma, a cancer with high TMB because of UV exposure, responds well to current immune checkpoint blockade immunotherapy (7). Consequently, the use of immunotherapy to treat low TMB cancers has been an unmet need. While classic immune checkpoint inhibition activates the natural immune response against cancer, more recently, there has been some success with adoptive T-cell therapy (ACT) that creates a repertoire against “nonself” neoantigens or tumor-associated antigens (*e.g.*, cancer germ line [CG] antigens) (8, 9). Thus, the identification of cancer-specific or cancer-associated antigen-derived peptides is important for the development of immunotherapeutic strategies for the treatment of low TMB tumors.

Recently, MS-based proteomics has become a powerful approach for large-scale profiling of the human leukocyte antigen (HLA) class I-presented peptidome (10, 11). Unlike traditional HLA-epitope prediction algorithms, MS sequencing provides direct experimental evidence of the presented peptides, and it allows for the relative quantification of cell surface peptide presentation. This high-throughput method can be used to profile thousands of *in vivo* HLA-associated immunopeptides (12, 13). When combined with next-generation sequencing (NGS), to reveal somatic mutations, this approach is capable of detecting mutant peptides (14) and noncanonical peptides derived from noncoding regions (15). However, despite the fact that MS-based cancer antigen discovery has been widely employed for directly assessing antigen presentation, many previous studies only focused on high TMB tumors. Here, our goal was to develop a comprehensive proteogenomic platform to identify potentially targetable class I-presented peptides in both melanoma and lung adenocarcinoma. We hypothesized that both low TMB-associated EGFR-mutant lung tumors and high TMB-associated melanoma present a repertoire of tumor-specific or tumor-associated antigen-derived peptides on HLA class I.

To develop this method, we enriched the cell surface–presented HLA class I-bound peptides in two primary melanoma cell lines, two EGFR-mutant lung adenocarcinoma cell lines, and one primary tumor from an EGFR-mutant patient who had undergone EGFR tyrosine kinase inhibitor therapy and identified those peptides using high-resolution tandem MS (MS/MS). Our datasets contain five major categories of class I-presented peptides derived from (1) common driver oncogenes; (2) mutated peptides derived from variants, herein, referred to as variant peptides; (3) CG antigen–derived peptides; (4) post-translationally modified (PTM) peptides; and (5) long noncoding RNA (lncRNA)–derived peptides. We also validated select class I-presented peptides utilizing synthetic standard peptides and an HLA stability–binding assay.

## Experimental procedures

### Human subject and cell lines

Lung tumor specimen was obtained at rapid autopsy from left lower lobe of the lung of osimertinib-treated patient NCI-RA007, a 70-year-old male, with a primary EGFR^L858R^ mutation. The tumor is a mixture of tumor cells and surrounding immune cells and fibroblasts that constitute the tumor microenvironment. The tumor specimen was snap frozen in liquid nitrogen upon collection. The genomic alterations in the tumor of this patient were described before (16). In accordance with the Declaration of Helsinki, the patient was consented with National Cancer Institute (NCI) Institutional Review Board–approved protocol 13-C-0131 (NCT01851395) entitled “A pilot study of inpatient hospice with procurement of tissue on expiration in thoracic malignancies.” The patient was offered hospice treatment with life expectancy less than 3 months at Center for Cancer Research (CCR), NCI. The rapid autopsies were initiated within 3 h upon patient death that resulted in good quality genomics and proteomics data from tumor tissues as reported before (17). The two primary melanoma cell lines, NCI-3784Mel and NCI-3795Mel, were obtained from patients with melanoma treated at the NCI, under the protocol, NCT00068003. NCI-3784Mel has been reported previously (18). The melanoma cells were cultured in high glucose Dulbecco's modified Eagle's medium supplemented with 20% fetal bovine serum (FBS). EGFR-mutant lung adenocarcinoma cell line H1975, was purchased from American Type Culture Collection (ATCC) and PC9 cell line was obtained from the Varmus Laboratory (MSKCC). The lung adenocarcinoma cells were maintained in RPMI1640 cell growth medium supplemented with 10% FBS. The T2 (174 × CEM.T2) cell line, purchased from ATCC, was maintained in ATCC-formulated Iscove's modified Dulbecco's medium supplemented with 10% FBS.

### HLA class I-presented peptide enrichment and purification

For HLA class I-presented peptide enrichment of the melanoma and lung adenocarcinoma cell lines, 2.0 × 10^8^ cells per biological replicate were harvested in 4 ml ice-cold lysis buffer (20 mM Tris–HCl, pH = 8.5, 100 mM NaCl, 1 mM EDTA, and 1% Triton X-100 supplemented with Halt 1:100 protease inhibitor cocktail [catalog no. 78430; Thermo Scientific]). After 30 min on ice, lysates were subjected to needle sonication for 30 s. For the human tumor tissue from rapid autopsy, 30 mg snap-frozen lung tumor tissue per replicate was homogenized in 4 ml ice-cold lysis buffer for 30 s at 4 °C using the Qiagen TissueLyser II. Cell/tissue lysates were centrifuged at 20,000*g* for 2 h at 4 °C, and the supernatant was used in subsequent experiments. HLA–peptide complexes were isolated by immunoprecipitating with 0.5 mg pan-HLA class I antibody clone W6/32 (BioXcell) precoupled with 200 μl slurry of protein A/G PLUS agarose resin (Santa Cruz Biotechnology) overnight at 4 °C with constant rotation. Agarose beads were then washed three times with ice-cold lysis buffer (without Triton and protease inhibitors), followed by two washes in ice-cold 20 mM Tris–HCl (pH = 8.5), and then one wash in ice-cold HPLC grade water. Complexes were eluted four times with 0.15% TFA in water at room temperature and combined. To purify immunopeptides, 50 mg C_18_ desalting columns (Sigma Millipore) were activated by two washes with 100% acetonitrile (ACN) and equilibrated by two washes with 0.1% TFA in water. HLA–peptide complexes were loaded on the preconditioned 50 mg C_18_ columns followed by three 0.1% TFA in water washes. HLA peptides were eluted with 40% ACN in 0.1% TFA. Purified peptides were lyophilized at −80 °C for 2 h followed by desalting step using pierce C_18_ Spin tips (catalog no. 84850; Thermo Scientific). The spin tips were activated using 20 μl of 80% ACN in 0.1% TFA and equilibrated using 20 μl of 0.1% TFA by centrifuging at 1000*g* for 1 min. We loaded the acidified HLA peptides (reconstituted in 40 μl of 0.1% TFA) on to prepared spin tips, washed the peptides twice with 0.1% TFA, and eluted the peptides in 20 μl of 80% ACN in 0.1% TFA. The resulting peptides were lyophilized and reconstituted in 0.1% TFA and 2% ACN loading buffer for LC–MS/MS analysis.

### Sample preparation of whole-cell proteomic profiling

The sample preparation protocol of H1975 whole-cell proteome profiling has been described previously (19). Briefly, three of 10-cm cell culture plates of H1975 (n = 3) were harvested using the same lysis buffer as for HLA peptide purification. About 1 mg protein was reduced by 5 mM Tris (2-carboxyethyl) phosphine hydrochloride at 50 °C for 30 min and was alkylated by 10 mM iodoacetamide in dark for 30 min followed by MS grade trypsin/lysC digestion (Promega) for 16 h at 37 °C. The resulting tryptic peptides were fractionated by an off-line high-pH (pH = 8.5) reverse-phase LC into a 96-well deep plate and were further pooled into 12 fractions. The LC mobile phase A was 10 mM triethylammonium bicarbonate in water, and the LC mobile phase B was 10 mM triethylammonium bicarbonate in ACN. The lyophilized tryptic peptides were reconstituted in phase A and separated with a 30 min LC gradient (10–35% solvent B) on an XBridge C18, 100 × 2.1 mm analytical column (Waters) using a flow rate at 0.25 ml/min. A total of 96 fractions were collected and acidified to 0.5% TFA and pooled into 12 final fractions immediately. The fractionated peptides were lyophilized and desalted by Pierce C_18_ Spin tips according to the described protocol for HLA peptides desalting. Purified peptides were reconstituted in 0.1% TFA and 2% ACN buffer, and peptide concentration was evaluated by NanoDrop at an absorbance at 215 nm. About 500 ng per fraction was subjected for LC–MS/MS analysis.

### MS/MS analyses

For the HLA immunopeptidome profiling, the purified and desalted HLA peptides were loaded to a 2 cm nano Acclaim trap column (catalog no. 164535) followed by separation on a 25 cm EASY-spray reverse phase column (catalog no. ES802A) for 90 min effective gradient with 4 to 35% 0.1% formic acid in ACN on an Ultimate 3000 Nano LC instrument (Thermo Scientific). The separated peptides were analyzed on an Orbitrap Q-Exactive HF mass spectrometer (Thermo Scientific) with discovery mode for data acquisition. The MS1 full scan (375–1650 *m/z*) was set to 120,000 resolution, and top 15 most abundant peptides per cycle were subsequently fragmentated by high-energy collision dissociation. To identify only nontryptic-digested neutral HLA class I-presented peptides (*e.g.*, without either lysine or arginine), typically containing 8 to 14 amino acid residues, we also included single charged ions resulting in charge state 1 to 4 for MS2 peak picking. The MS2 sequencing scans acquired the peptide fragments at 30,000 resolution and 200 ms maximum injection time window. Since enriched HLA peptidome is commonly less complex than whole-cell proteome, we used dynamic exclusion at 20 s to collect more MS1 data points, allowing better peak area–based quantitation. For the whole-cell proteome, H1975 tryptic peptides from each fraction were separated by the same LC method described previously for the HLA peptidome profiling. The MS1 analysis was set at 60,000 resolution, and top 20 most abundant peaks were selected for MS/MS analysis in which we used 15,000 resolution and 32 ms maximum injection window. We included charge state 2 to 6 for MS2 fragmentation, and dynamic exclusion was set at 30 s. In addition, we validated select variant peptides, CG antigen and lncRNA-derived peptides. Select variant peptides and lncRNA-derived peptides were *in vitro* synthesized by GenScript. Select melanoma CG antigen–derived peptides were *in vitro* synthesized by the Peptide Synthesis and Antigen Discovery Core, Surgery Branch, NCI. Synthetic peptides were serial diluted to 1 pmol/μl in 0.1% TFA and 2% ACN in water. The diluted synthetic peptides were pooled, and 5 μl of the pool was subjected to MS/MS analysis using the same LC–MS/MS instrument method for HLA peptidome profiling. To correct potential retention time (RT) shifting using different batches of the nanoLC columns, Pierce Peptide Retention Time Calibration Mixture (Thermo Fisher; catalog no. 88321) containing 15 heavy-labeled peptides (1 pmol/μl) was used for the RT correction.

### Database and *de novo* search of MS raw files

Patient- and cell line–specific protein sequence databases (DBs) were first generated. The aligned whole-exome sequencing (WES) BAM files from patient blood (germ line) or tumor tissue/cell lines were used to retrieve the variant call format (VCF) files using HaplotypeCaller (Broad Institute) (20), and the intermediate VCF files were further annotated by SnpEff (Microsoft Genomics) (21), which filtered out only nonsynonymous variants on exome regions including single nucleotide variants (SNVs) and small insertions and deletions (INDELs). Similarly, BBduk (v35.2) (DOE Joint Genome Institute) was used to remove adapter sequences and low-quality reads from paired-end FASTQ files, which were then used as input for STAR-Fusion (version 1.6.0) (https://github.com/STAR-Fusion/STAR-Fusion/) (22). The final VCF files were *in silico* translated to sample-specific protein sequence libraries (FASTA files) using QUILTS (http://openslice.fenyolab.org/cgi-bin/pyquilts_cgi.pl) (23), which were merged with refseq hg38 converted human proteome DB (v20130727). Herein, we generated specific FASTA file for each patient and cell line. To avoid using hypercomplex DB, which may increase the false-positive rate, DB search for variant peptide discovery was carried out for individual tumor or cell line separately. For the identification of nonvariant peptides (*i.e.*, CG antigen and PTMs), DB search was conducted with all MS raw files using UniProt human proteome DB (v20170207). The DB search of MS raw files was carried out by PEAKS studio (version 8.5) (24) (Bioinformatics Solutions) using the patient-/cell line–specific DBs or standard human proteome DB described previously. In the PEAKS search engine, no enzyme digestion was selected because HLA peptides are natural peptides without artificial enzyme digestion. Importantly, the unique PEAKS built-in functions, pan-PTMs including 650 different variable modifications and *de novo* search algorithm, were used. The precursor mass tolerance was set to 15 ppm, and the fragment ion tolerance was set to 0.05 Da. For the DB search, the false discovery rate (FDR) of peptide identification, estimated by decoy-fusion DB, was chosen at 0.01. For the *de novo* search, the average local confidence (ALC%) score of each peptide was chosen to be >50%. MS1 peak area–based label-free quantitation method was used for peptide quantification. The direct output peptide intensity from PEAKS was log2 transformed for further statistical analysis. For the whole-cell proteome, 12 fractions from each sample were pooled, and DB was searched by MaxQuant (version 1.5.7.4) (Max Planck Institute) using UniProt human proteome DB (v20170207) that contains 70,948 entries including isoforms. The mass tolerance for precursor ions was set to 4.5 ppm, and mass tolerance for fragment ions was set to 20 ppm. Trypsin and lysC were selected as digestion enzymes, maximum missed cleavage was set to 4, and methionine oxidation and N-terminal acetylation were selected as variable modifications. Both FDRs at peptide and protein levels were set to 0.01. The proteins were quantified by label-free quantification method.

### HLA class I typing

**Supplemental Figure S1:**
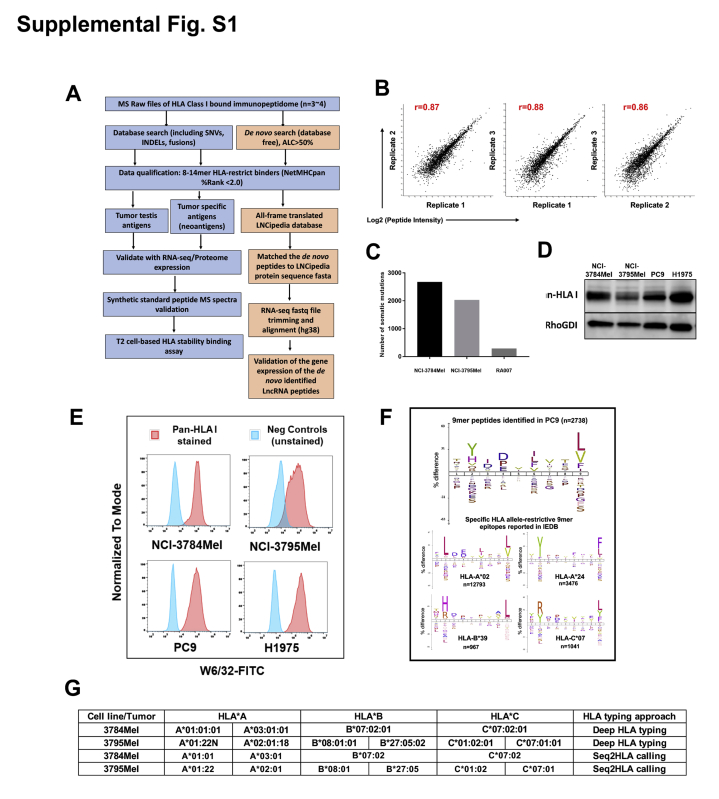


First, HLA typing (six digits) of the patient donors of the two primary melanoma cell lines was performed using sequence-specific primer (SSP) and Sanger sequencing technologies by the Department of Transfusion Medicine of Clinical Center at National Institutes of Health (NIH). In addition, our laboratory conducted four-digit HLA class I calling from the WES data of these two melanoma cells using Seq2HLA package (25) and demonstrated consistent results with the SSP-Seq (supplemental Fig. S1*G*) Therefore, we performed Seq2HLA-based informatic HLA typing from the WES results of all samples.

### T-cell epitopes/HLA-binding prediction, motif analysis, and hydrophobicity index prediction

All peptides except PTM peptides in each cell/tissue sample were used for T-cell epitope and HLA-binding prediction using Immune Epitope Database and Analysis Resource (IEDB) (26) and/or NetMHCpan-4.0 (http://www.cbs.dtu.dk/services/NetMHCpan-4.0/) (27). To generate reviewed monoallelic epitope datasheets using IEDB (supplemental Fig. S1*F*), we applied stringent filters for those epitopes: (1) “*Homo sapiens*” as antigen organism; (2) “Humans” as host; and (3) specific HLA class I subtype (*e.g.*, HLA-A∗02). The resulting known epitopes from IEDB DB were subject to motif analysis using iceLogo (https://iomics.ugent.be/icelogoserver/) (28), where we used *H. sapiens* SwissProt composition as the reference set, and start position was set to 1. The percent difference in frequency of the amino acid at given locations was chosen as the readout of motif analysis. To compare the motifs from those reported epitopes and MS-detected HLA immunopeptides, we performed similar motif analyses of our peptide datasets. The hydrophobicity index (HI) prediction of select *de novo* peptides was performed in SSRCalc (version Q) where 100 Å C18 column, 0.1% formic acid (2015), and HI_(Best)_ were selected (29).

### Whole exome and total RNA-Seq

WES and total RNA-Seq were performed as described previously (17). Briefly, the genomic DNA and total RNA of the cell lines and tumor were extracted and sent to the NGS core facility at NCI Frederick National Laboratory. The samples were sequenced as 2 × 126 nt paired end reads with Illumina HiSeq2500 sequencers with >100 million reads per sample. The raw FASTQ files were aligned to hg38 by TopHat (version 2.0.13) (https://github.com/infphilo/tophat) (30); the aligned BAM files were used for downstream variant calling. For the samples with a corresponding germ line specimen, Strelka (version 1.0.10) was used for somatic variant calling (31). The total RNA-Seq of H1975 (n = 3) was normalized and quantified using DESeq2 (version 1.30.0) (32).

### Generation of CG antigen DB

We compiled a CG antigen DB using existing antigens reported in CT DB (http://www.cta.lncc.br/), Human Protein Atlas (https://www.proteinatlas.org/), and Cancer Antigenic Peptide DB (https://caped.icp.ucl.ac.be/).

### Immunoblotting of HLA class I antigens

One million cells from each cell line were lyzed in ice-cold modified radioimmunoprecipitation buffer for 30 min. The cell lysate was spun down at 20,000*g* for 15 min at 4 °C, and supernatant protein concentration was determined by the bicinchoninic acid protein assay. About 10 μg of protein from each cell line underwent SDS-PAGE. Subsequently, separated proteins were transferred to polyvinylidene fluoride membrane and incubated with primary anti-HLA class I mouse horseradish peroxidase monoclonal antibody at 1:5000 dilution (EMR8-5; Funakoshi) overnight at 4 °C, and then briefly incubated with SuperSignal horseradish peroxidase substrates (Thermo Scientific) before imaging. The membranes were exposed for 5 s, and images were acquired by Odyssey Fc imager (LI-COR Biosciences).

### Flow cytometry analyses for HLA class I expression

One million cells were collected in 100 μl fluorescence-activated cell sorting buffer (PBS + 5% FBS). After 30 min of blocking, half of the cells were incubated with FITC anti-HLA class I, W6/32 (catalog no. 311404; BioLegend) for 30 min at 4 °C, and washed twice with fluorescence-activated cell sorting buffer. Flow cytometry was carried out on CytoFLEX platform (Beckman), and 20,000 events were collected per sample. The postanalyses and statistics were conducted by FlowJo (version 10.6.2) (BD Biosciences).

### HLA-binding affinity T2 cell assay

The antigen peptide transporter (transporter associated with antigen processing [TAP])–deficient HLA-A2 only expressing T2 cells presents lower affinity peptides because of TAP1 deficiency and allow for an efficient exchange of high-affinity peptides. We suspended T2 cells at 1.0 million/ml in growth medium and plated them in 6-well tissue culture plates (2 ml/well). The synthetic peptides for validation and NY-ESO-1 peptide (positive control) were reconstituted in dimethyl sulfoxide (DMSO) and diluted to a final concentration of 10 μM with growth medium and incubated with T2 cells at 37 °C with 5% CO_2_ for 12 h. Each testing peptide and positive control was performed in triplicate and negative control (DMSO) in duplicate. The cells were incubated with FITC anti-HLA class I, W6/32 (catalog no. 311404; BioLegend) for 30 min at 4 °C followed by two washes. Flow cytometry was performed to detect the cell surface total HLA class I protein expression. Data analysis was conducted by FlowJo (version 10.6.2), and geometric mean fluorescence intensity was used for quantification.

### Identification of HLA-presented lncRNA-derived peptides

To identify lncRNA-coded peptides, we generated an lncRNA-translated protein sequence DB, which was *in silico* 6 frame translated from a high-confident lncRNA DB, LNCipedia (www.lncipedia.org), containing 107,039 lncRNA transcripts from 49,372 annotated distinct lncRNAs (version 5.2) (33). This resulted in 642,234 lncRNA-encoded protein sequence entries. The *de novo*–only identified 8- to 14-mer peptides that did not match the normal Uniprot human DB (70,948 entries including isoforms) from all five samples were queried against this lncRNA DB-derived six-frame translated protein sequence DB. Furthermore, we only kept the peptides predicted to be an HLA binder (%Rank <2.0) to at least one HLA allele in respective samples. To ensure those matched lncRNA-derived peptides were truly expressed at RNA level, we generated intersect BED files between each sample's total transcriptome and LNCipedia. Briefly, for the genomic data processing, quality control was done on total RNA-Seq FASTQ files by first assessing read quality using FASTQC (version 0.11.8) followed by removing adapter sequences and low-quality reads using BBduk, part of the BBTools package (version 38.42). The resulting FASTQ files were aligned against the RefSeq human genome version hg38 using STAR (version 1.3.4) (34) and sorted using the Samtools mappings sorter (version 1.1.1) (35). Duplicate reads were then removed using Picard MarkDuplicates (version 2.1.1). These alignments, sorting, and deduplication steps were run on the DNANexus platform. The resulting BAM file was converted to BED format using the bamtobed tool from the BEDTools suite (version 2.29.0) (36). The BEDTools intersect tool was used to find the intersection between the tumor or cell-line BED file and the LNCipedia BED file (version 5.2). The resulting BED files contained matched expressed lncRNA genes in the sample transcriptome. Because of the possible misannotated lncRNAs in LNCipedia DB, we further visualized each individual matched lncRNA using the intersect BED files in Integrative Genomics Viewer (IGV; version 2.5.3) (https://software.broadinstitute.org/software/igv/) (37). Furthermore, each peptide-coding region was identified using BLAST-like Alignment Tool (BLAT) in IGV. Any peptide whose corresponding matched lncRNA also matched to the noncoding region or intron region, but not the coding exon region of the human genome, was kept as potential lncRNA-derived peptides. For the ribosome sequencing, we utilized a well-recognized ribo-seq genome browser (https://gwips.ucc.ie/index.html), which compiles 46 ribosome profiles (38). The lncRNA containing ORFs were manually searched on the Genome-Wide Information on Protein Synthesis (GWIPS) to confirm the lncRNA-derived peptide-coding regions RNAs were bound to ribosome.

For the empirical *p* value evaluation, 50,000 nonoverlapping genomic segments, each of length 2000 nucleotides (the mean length of lncRNAs in the LNCipeida DB), were sampled uniformly at random from the human reference genome sequence. These random genomic segments, similar in size to LNCipedia, were used as a “mock”/decoy lncRNA DB. Subsequently, an *in silico* translation of these mock/decoy lncRNA sequences was performed *in silico*, using each of the six possible reading frames, to generate a mock/decoy protein sequence DB. Of the entire set of peptides identified by the PEAKS *de novo* algorithm within class I immunopeptidome, 66 had a match in this mock/decoy protein DB. For obtaining a match, the translation of each of the six potential reading frames for every mock/decoy lncRNA sequence was considered. For obtaining a rather pessimistic estimate (*i.e.*, larger than the correct value) on the *p* value for the actual number of matches between the peptides identified by the PEAKs *de novo* algorithm and the LNCipedia DB, we set the probability of a chance match of a peptide (which, on average, has 11 amino acids, coded by 33 nucleotides) to *p* = 66 matches/(50,000 transcripts × 6 reading frames × 2000 nucleotides per transcript/33 nucleotides per peptide) = 3.63e^−6^ (this forms the null assumption). LNCipedia DB includes 107,039 lncRNA transcripts from 49,371 distinct lncRNAs. The number of *de novo* peptides identified by the PEAKS *de novo* algorithm within class I immunopeptidome, which had a match in the LNCipedia DB, was 195. The probability of obtaining exactly k matches among 107,039 lncRNA transcripts in the LNCipedia DB, each giving rise to (6 × 2000)/33 potential peptides can be calculated using the aforementioned *null assumption* as

(1) $$q_{k}=\left(\begin{array}{l} n\\ k \end{array}\right)p^{k}{\left(1-p\right)}^{\left(n-k\right)}$$

where n = (107,038 × 120,000)/33. The empirical *p* value for having 195 matches for class I immunopeptidome among the LNCipedia DB entries can thus be calculated as:

(2) $$p\text{ value}=\sum_{k=195}^{k=n}q^{k}$$

The lncRNA-derived immunopeptidome bioinformatics analysis was performed on the Biowulf Linux cluster at the NIH (https://hpc.nih.gov/docs/userguide.html). The “mock/decoy” DBs, empirical *p* value calculation method, and the original Python scripts of the identification of HLA-presented lncRNA-derived peptides can be found at Github (https://github.com/YueAndyQi/lncRNA_immunopeptidome_Scripts).

### Functional annotation of source proteins of HLA immunopeptidome

We unitized ingenuity pathway analysis (IPA) to determine the subcellular localization/molecular function and cell signaling pathway analysis of the source protein of identified HLA peptides (39). Furthermore, we identified the upstream transcriptional modulators of those source proteins using the unique upstream regulator analysis feature in IPA.

### Experimental design and statistical rationale

Two EGFR-mutant lung adenocarcinoma cell lines, PC9 and H1975, harboring EGFR^Del E746–A750^ and EGFR^L858R/T790M^, respectively, were used for this study representing low TMB tumors. To apply the comprehensive HLA immunopeptidome discovery pipeline in human tumor tissue, a lung tumor harboring EGFR^L858R^ that was procured at rapid autopsy from a patient (NCI-RA007) treated with the third-generation EGFR tyrosine kinase inhibitor, osimertinib, was selected. In addition, two melanoma cell lines, NCI-3784Mel and NCI-3795Mel, generated from tumors of patients with melanoma treated at the NIH Clinical Center, which represent tumors with high TMB. We identified somatic mutations by WES of tumor or tumor-derived cell lines and corresponding germ line DNA from patients NCI-RA007, NCI-3784Mel, and NCI-3795Mel. Expressed somatic mutations were also identified by RNA-Seq of the cell lines and tumor. PC9 and H1975 lung adenocarcinoma cell lines did not have corresponding germ line DNA; hence, all mutations identified by WES and RNA-Seq were included for our data analyses. The identified mutations included SNVs, small INDELs, and fusions. The pan-HLA class I antibody was used to immunoprecipitate class I proteins along with their presented peptides that were sequenced by high-resolution MS/MS. The MS data were searched against cell line– or tumor-specific peptides created by adding all corresponding mutant peptides to the normal human DB. The MS data were also searched using the *de novo* search algorithm in PEAKS studio. To assess experimental reproducibility and perform statistical tests, three biological replicates were performed for PC9, H1975, and NCI-3795Mel and four biological replicates for NCI-3784Mel and NCI-RA007. Each biological cell line replicate was initiated from the same cell passage number but cultured in separated dishes. Four biological replicates of the tumor specimen NCI-RA007 were obtained by sampling four regions of the tumor obtained at rapid autopsy. The two-way *t* test and ANOVA test were applied to two-group comparison or more than two-group comparisons, respectively. Finally, since conventional decoy protein sequence–based FDR assessment is not applicable for the PEAKS *de novo* search results, we calculated empirical *p* value by creating a mock lncRNA DB to control the random matches and false positives for our lncRNA-derived peptide identification.

## Results

### Identification and characteristics of the HLA class I-presented immunopeptidome and HLA class I expression in melanoma and lung adenocarcinoma

**Fig. 1:**
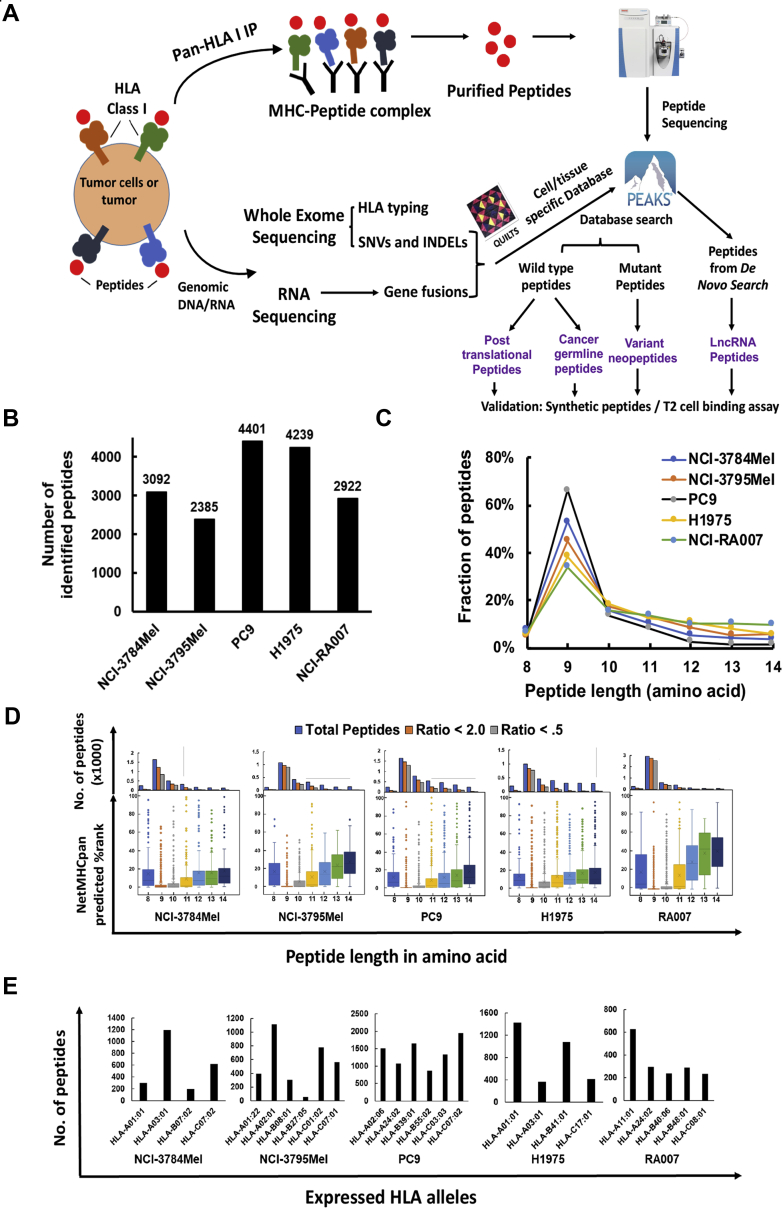
**Overview of HLA class I-presented immunopeptides identified from EGFR-mutant lung cancer and melanoma patient–derived cell lines by MS.***A*, strategic plan of proteogenomic analysis pipeline for HLA class I immunopeptidome using MS-based proteomics and next-generation sequencing. *B*, total number of peptides identified in the HLA class I immunopeptidomes from patient-derived melanoma and EGFR-mutant lung cancer cell lines and tumor. *C*, the peptide length distribution within the class I immunopeptidome from all samples. *D*, NetMHCpan 4.0 prediction algorithm-based scoring of each MS-identified peptide and distribution of binding scores among 8- to 14-mer peptides. *Upper panel* shows the distribution of total identified peptides, binders (%Rank <2.0), and strong binders (%Rank <0.5) for each peptide length. *Lower panel* shows box plots of the lowest NetMHCPan predicted %Rank for binding among the corresponding HLA class I alleles for each peptide identified for various peptide lengths. *E*, number of predicted binders (%Rank <2.0) assigned to different HLA alleles for each sample based on its corresponding HLA class I alleles. EGFR, epidermal growth factor receptor; HLA, human leukocyte antigen.

**Supplemental Figure S2:**
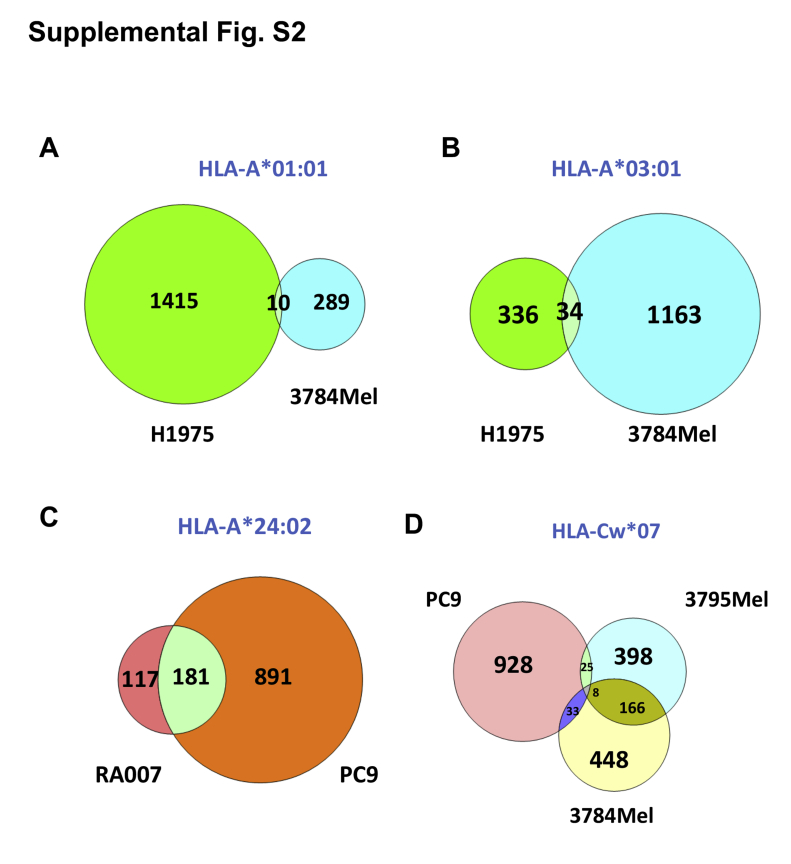


We identified 35,233 HLA class I-presented peptides containing 8 to 14 amino acid residues empolying various experimental and informatic tools including pan-HLA class I immunoprecipitation, elution of the class I-presented peptides, high-resolution MS-based peptide sequencing, NGS of genomic DNA/RNA to create patient- and cell line–specific DBs incorporating variant peptides, and computational algorithms (Fig. 1*A*). These include 14,876 DB-searched peptides and 20,357 *de novo* sequencing algorithm–searched peptides (supplemental Fig. S1*A* and supplemental Table S1). We performed three to four biological replicates of HLA class I pull-down experiments and MS analyses of associated peptides from each cell line/tumor. Pairwise correlation coefficients of peptide intensities from three representative biological replicates from PC9 cells show relatively high correlation between replicates (supplemental Fig. S1*B*). We identified 2385 to 4401 DB-matched peptides in each sample. Interestingly, the number of HLA class I-presented peptides identified in melanoma and EGFR-mutant lung cancer was roughly similar (Fig. 1*B*). More peptides were identified from the lung adenocarcinoma cell lines, PC9 and H1975, compared with the EGFR-mutant tumor, NCI-RA007, and the melanoma patient–derived cell lines. A majority of enriched immunopeptides were 9-mer (Fig. 1*C*), consistent with the length of HLA class I-bound immunopeptidomes reported previously (13, 40). We further analyzed our dataset using NetMHCpan by which we determined weak and strong binding using %Rank <2.0 and <0.5, respectively. A majority of the enriched peptides were predicted to be binders; nonetheless, 9-mer and 10-mer peptides had lower scores and hence predicted stronger binding (Fig. 1*D*). The predicted HLA binders were assigned to the expressed HLA alleles in each sample (Fig. 1*E*). Somatic mutations were identified using WES of tumor and germ line DNA; as expected, EGFR-mutant lung adenocarcinoma patient NCI-RA007 had much fewer somatic mutations (289) compared with the melanoma patient–derived cell lines, NCI-3784Mel and NCI-3795Mel (2678 and 2031, respectively) (supplemental Fig. S1*C* and supplemental Table S2). The differences in abundance of class I-presented peptides may be a result of the expression level of HLA class I proteins. The total HLA class I protein expression detected by immunoblotting (supplemental Fig. S1*D*) was consistent with cell surface HLA class I presentation analyzed by flow cytometry (supplemental Fig. S1*E*) and was approximately similar between the melanoma and the lung adenocarcinoma cell lines. Since HLA proteins are highly polymorphic and the peptides presented are HLA allele restrictive (41, 42), HLA typing is important to further characterize the HLA class I-presented peptides identified. Tumors and cell lines were HLA typed using seq2HLA analysis (25) of the WES data (Table 1), and our data suggest that seq2HLA produced consistent results with conventional HLA typing using SSP sequencing in the two melanoma cell lines (supplemental Fig. S1*F*). It is interesting to note that NCI-3784, H1975, and NCI-RA007 had loss of heterozygosity of *HLA-B* and/or *HLA-C* alleles. HLA loss of heterozygosity, often caused by genetic alterations of chromosome 6p (43), has been suggested as a mechanism of immune escape (44). Furthermore, we validated our peptidome using previous reported and/or experimentally validated binding peptidome from the IEDB. Since 9-mer is the most common length for peptides in the HLA class I immunopeptidome, we visualized the 9-mer peptide–binding motifs of our enriched HLA class I peptidome from tumor cell lines/tissue and their corresponding monoallelic datasheets from IEDB. For example, HLA–peptide binding motifs of four major HLA-typed alleles in PC9 cells are the main components of endogenous enriched PC9 immunopeptide by overlaying the four-individual monoallelic binding motifs (supplemental Fig. S1*G*). Computational HLA-binding epitope prediction algorithms have been widely recognized as a powerful tool to estimate HLA-ligand binding affinity and tumor neoantigen prediction (45). Notably, although A∗01:01 and A∗03:01 were both present in NCI-3784mel and H1975, they barely share any binding peptides, suggesting the possibility of different HLA ligand processing and presentation machinery and/or different source protein expression levels in melanoma and lung cancer (supplemental Fig. S2, *A* and *B*). We observed a similar phenomenon in HLA-A∗24:02 (typed in PC9 and NCI-RA007) and Cw∗07 (typed in PC9, NCI-3784Mel, and NCI-3795Mel) (supplemental Fig. S2, *C* and *D*).

**Table 1:** HLA typing of cancer cell lines and lung adenocarcinoma tumor

Cell line/tumor	Origin	HLA-A		HLA-B		HLA-C	
NCI-3784Mel	Melanoma	A∗01:01	A∗03:01	B∗07:02		C∗07:02	
NCI-3795Mel	Melanoma	A∗01:22	A∗02:01	B∗08:01	B∗27:05	C∗01:02	C∗07:01
PC9	Lung tumor	A∗02:06	A∗24:02	B∗39:01	B∗55:02	C∗07:02	C∗03:03
H1975	Lung tumor	A∗01:01	A∗03:01	B∗41:01		C∗17:01	
NCI-RA007	Lung tumor	A∗11:01	A∗24:02	B∗40:06	B∗48:01	C∗08:01	

**Supplemental Figure S3:**
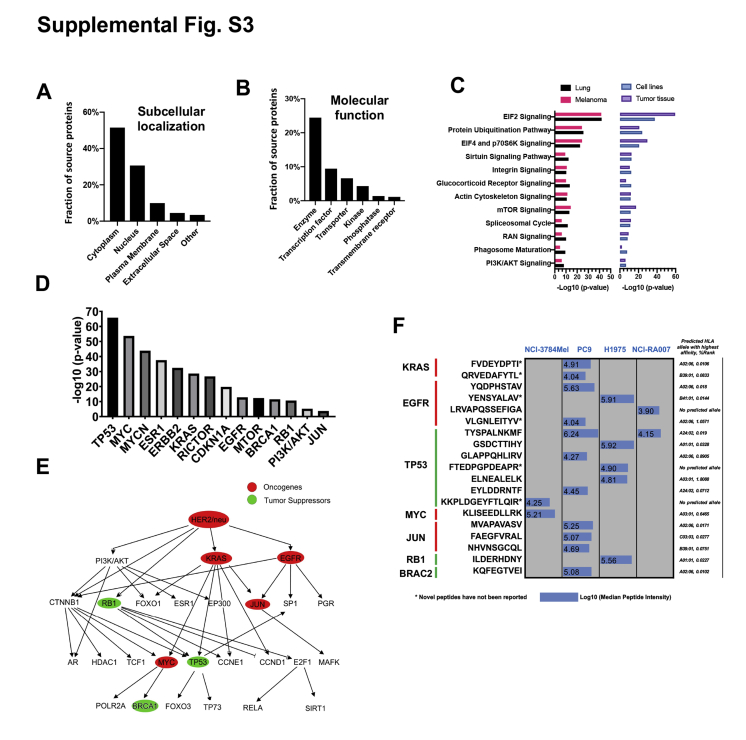


To annotate the source proteins of all class I-presented immunopeptides, we classified them based on their subcellular localization and molecular function (supplemental Fig. S3, *A* and *B*). A large majority of peptides identified were from cytoplasmic proteins and enzymes. Pathway analysis of identified parent proteins identified key pathway proteins contributing to class I immunopeptidome, such as eukaryotic initiation factor 2 (EIF2) signaling, protein ubiquitination pathway, EIF4 and p70S6K signaling, and others (supplemental Fig. S3*C*). IPA upstream regulator analysis showed tumor suppressor TP53 and proto-oncogenes *MYC*, *KRAS*, *ESR1*, *ERBB2*, *EGFR*, and *MTOR* to be among the top upstream potential regulators of the parent proteins identified (supplemental Fig. S3*D*). Network analysis confirmed that tumor suppressors (*e.g.*, TP53, BRCA1) and oncogenes (*e.g.*, EGFR, KRAS, MYC) were components of the network of parent proteins represented by the identified class I-presented peptides (supplemental Fig. S3*E*). To further supplement the bioinformatics analyses, we identified HLA class I-presented peptides from common proto-oncogenes, such as *KRAS*, *EGFR*, *MYC*, *JUN*, and tumor suppressors, such as *TP53*, *RB1*, and *BRCA2*. We identified 17 common cancer driver gene–derived wildtype peptides presented by HLA class I in lung adenocarcinoma cell lines/tumor and two peptides in primary tumor–derived melanoma cells; six of which are novel peptides not previously reported. In addition, we used NetMHCpan to predict the HLA class I restriction of the identified peptides from cancer drivers. We found that, though some peptides have been predicted to be binders for multiple respective HLA alleles (*e.g.*, KQFEGTVEI derived from BRCA2), their MS intensity is not significantly higher than that of the peptides predicted to be monoallelic binders (*e.g.*, KLISEEDLLRK derived from MYC) (supplemental Fig. S3*F* and supplemental Table S3). Taken together, our analyses identified 19 high-confidence oncogene/tumor suppressor–derived peptides that were presented by HLA class I.

### Discovery of variant class I-presented peptides in lung adenocarcinoma

**Fig. 2:**
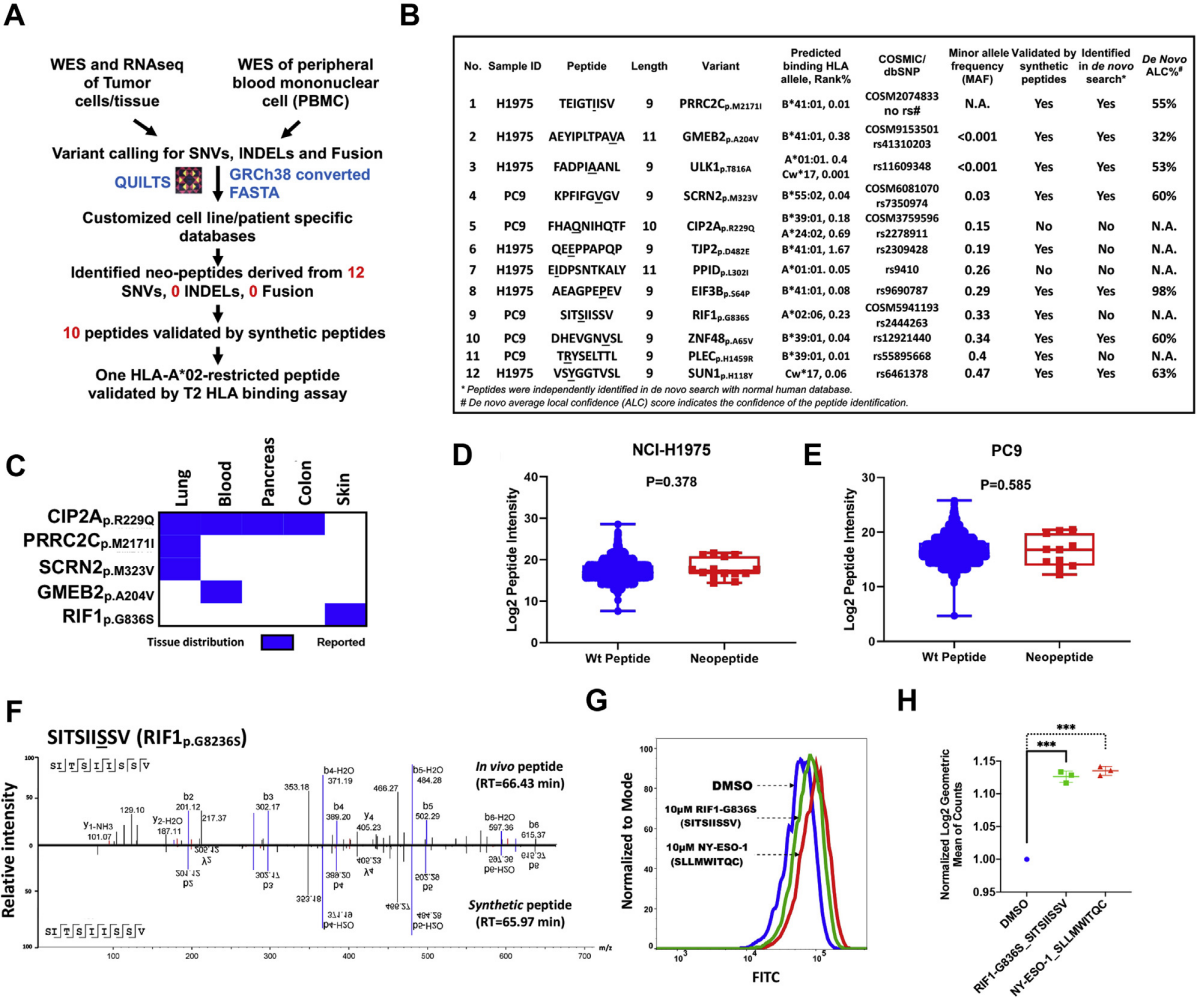
**Identification of class I-presented variant peptides in lung adenocarcinoma.***A*, workflow of integrated proteogenomic analysis using germ line (peripheral blood mononuclear cells) and tumor cell line/tissue WES and RNA-Seq datasets to identify SNVs, INDELs, and fusions and construction of tumor cell line–/tumor tissue–specific databases to interrogate the MS data of class I-associated peptides. *B*, list of 12 variant peptides, with the mutation underlined, predicted HLA restriction, dbSNP ID, synthetic peptide validation, and *de novo* sequencing search ALC score. *C*, tumor tissue distribution of five COSMIC-reported somatic mutations in the identified variant peptides. *D* and *E*, box plots show peptide intensity of wildtype and mutant peptides in all biological replicates from *C*, H1975 and *D*, PC9 cells. *F*, matched MS2 spectra of endogenous and its synthetic counterpart for RIF1-G836S-derived peptide SIT*S*IISSV. *G*, T2 cell-based HLA stability assay showing RIF1_p.G836S_-derived peptide (SIT*S*IISSV) bounding and stabilizing HLA-A∗02. *H*, box plot shows statistically significant increase of HLA expression (log_2_ geometric mean of counts) in T2 cells incubated with RIF1_p.G836S_-derived peptide (SIT*S*IISSV) and positive control NY-ESO1-derived peptide compared with those incubated with DMSO (*p* < 0.005). ALC, average local confidence; COSMIC, Catalogue of Somatic Mutations in Cancer; DMSO, dimethyl sulfoxide; HLA, human leukocyte antigen; INDEL, insertion and deletion; SNV, single nucleotide variant; WES, whole-exome sequencing.

Variant peptides derived from somatic mutations in tumors, if presented by MHC class I, have the potential to engage cytotoxic T cells to promote tumor immunity. Hence, the identification of variant peptides presented by the cognate class I proteins is of paramount importance. To identify mutant peptides, we first constructed cell line– and patient-specific DBs by adding all somatic variants identified by NGS (RNA-Seq and WES) to normal human DB used for searching MS data when germ line DNA was available for sequencing (NCI-3784Mel, NCI-3795Mel, and NCI-RA007) (Fig. 2*A*). For PC9 and H1975 cells, which do not have available germ line DNA, all variants identified by exome sequencing were used. We identified 12 peptides harboring SNVs in the two lung adenocarcinoma cell lines but no INDELs and fusions. Indeed, further analyses of these variant peptides using NetMHCpan predicted all to be binders for at least one HLA allele in their corresponding cell line (Fig. 2*B*). We leveraged dbSNP and Catalogue Of Somatic Mutations in Cancer (COSMIC) DBs to classify whether the peptides were derived from normal polymorphism or somatic mutations. We identified five mutations that were reported in COSMIC DB, and three of them have a minor allele frequency <0.05, confirming they are rare mutations. Next, we interrogated the tumor types in which the five variants have been identified. Indeed, proline-rich coiled-coil 2C (PRRC2C)_p.M2171I_ and secernin 2 (SCRN2)_p.M323V_ were exclusively discovered in lung cancer; importantly, cellular inhibitor of PP2A (CIP2A)_p.R229Q_ (legacy identifier: COSM3759596) has been confirmed as a somatic mutation in multiple cancers (*i.e.*, lung, blood, pancreas, and colon) according to COSMIC DB (Fig. 2*C*). Therefore, peptide FHAQNIHQTF derived from somatic mutation CIP2A_p.R229Q_ might be a potential CD8 T-cell target to a variety of cancer patients carrying this somatic mutation and corresponding HLA alleles (*e.g.*, A∗24:02 and/or B∗39:01).

**Supplemental Figure S4:**
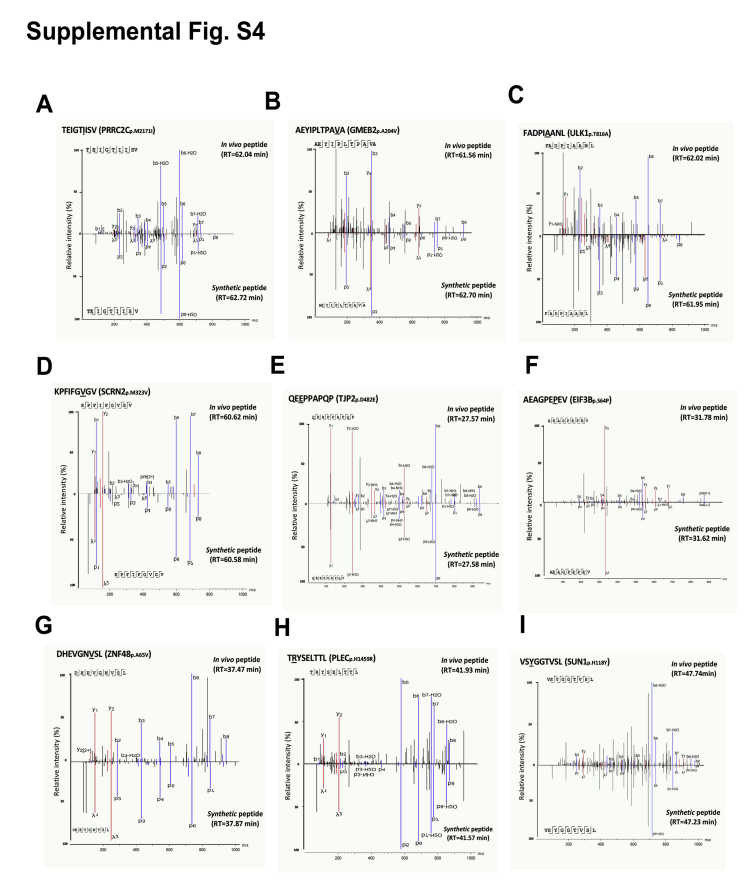


We compared the MS intensities of the 12 variant peptides and nonmutated class I-presented peptides and found no significant difference in median peptide intensities between the two groups for both the H1975 and PC9 cell lines (Fig. 2, *D* and *E*), suggesting that individual variant peptides are presented to similar extent as wildtype peptides. In addition, to validate the peptide sequences identified in our class I pull-down experiments, we synthesized a subset of the identified peptides and utilized LC MS/MS to compare the MS2 spectra identified for the synthesized and endogenous peptides. MS2 spectra and RT of ten of 12 synthetic peptides and their corresponding endogenous peptides were matched (Fig. 2*F* and supplemental Fig. S4, *A*–*I*). Next, we confirmed the binding of the peptides to specific predicted HLA alleles. Peptide SITSIISSV, derived from RIF1_p.G836S_, binds strongly to HLA-A∗02:06 with %Rank at 0.23. To assay cell surface HLA stabilization, we pulsed this peptide overnight to a TAP-deficient T2 cell line that expresses only HLA-A∗02 (46) and used HLA-A∗02 binding peptide SLLMWITQC from NY-ESO-1 as a positive control in parallel. Both peptides significantly stabilized HLA to the cell surface in comparison to the DMSO control (Fig. 2, *G* and *H*). Taken together, we identified 12 variant peptides in lung adenocarcinoma cell lines; notably, five of these were derived from reported somatic mutations. With spectra and HLA-binding validations, our results suggest that these variant peptides are potential targets for cancer immunotherapy.

### CG antigen–derived peptides presented by HLA class I in melanoma and lung adenocarcinoma

**Fig. 3:**
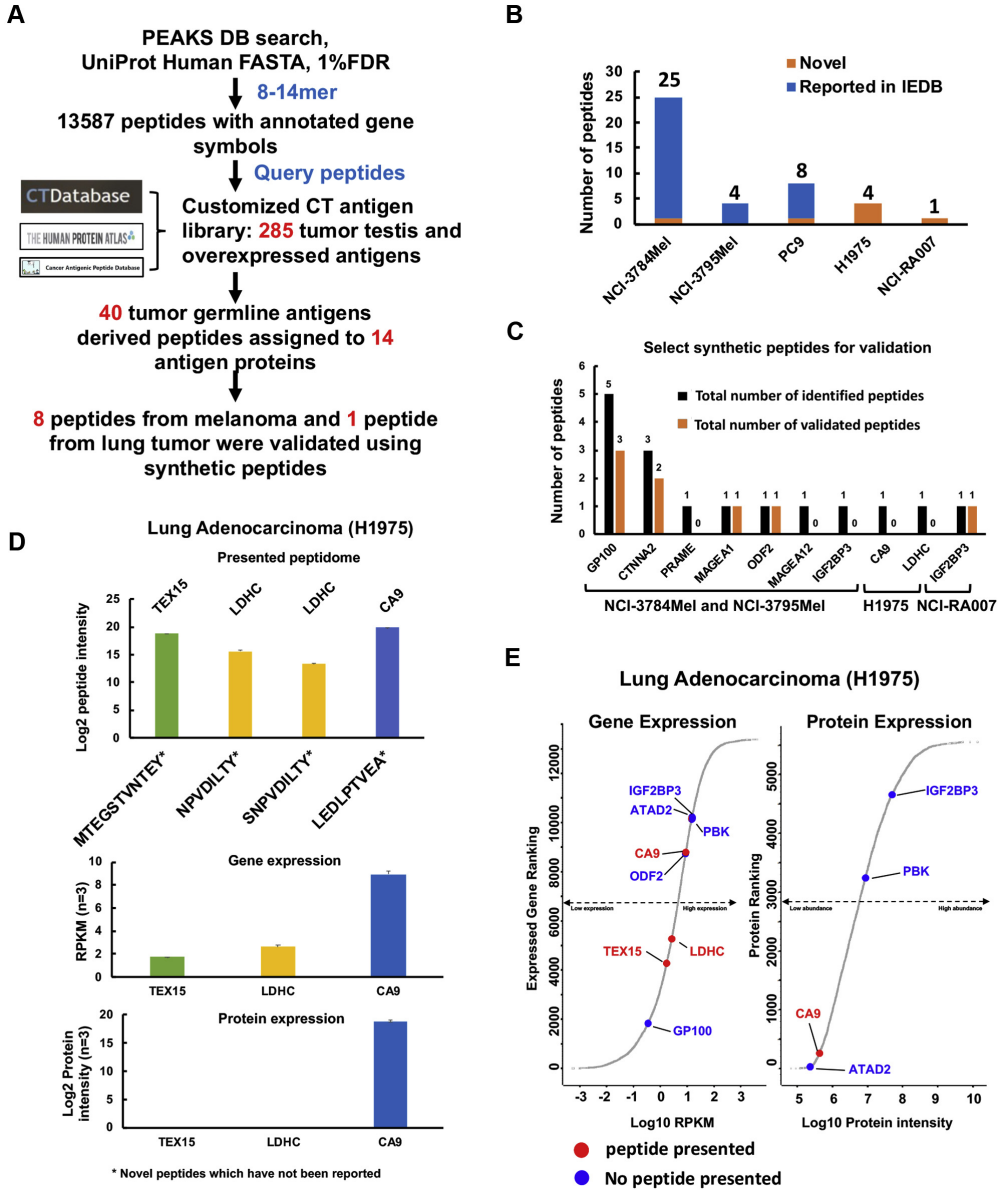
**Identification of cancer germ line antigen–derived peptides.***A*, overall strategy of CG antigen identification using customized library integrating previously reported immunogenic CG antigens; a total of 285 CG antigens were included in this custom database. *B*, the number of novel and reported CG antigen–derived peptides. *C*, summary chart of select peptides validated in NCI-3784Mel, NCI-3795Mel, H1975, and NCI-RA007 using synthetic peptide spectra match. *D*, log2 median peptide intensity of four immunopeptides identified in H1975 (*top panel*); gene expression (*middle panel*), and protein expression (*lower panel*) of three source antigen proteins. *E*, rank of transcript and protein expression of CG antigens identified in H1975 using RNA-Seq and MS, respectively. *Red dots* indicate CG antigens from which at least one class I-presented peptide was identified by MS. *Blue dots* indicate the CG antigens that were expressed in H1975 cells, but no class I-presented peptide was identified by MS. CG, cancer germ line.

**Supplemental Figure S5:**
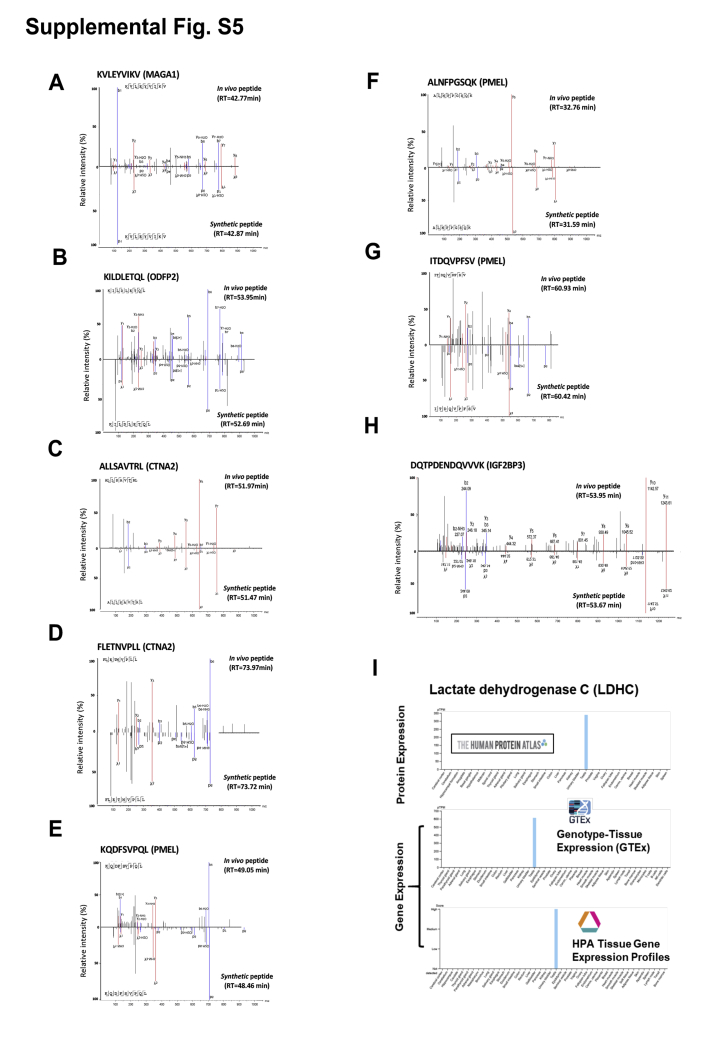


CG or tumor testis antigens hold great potential for generating tumor-specific antigens for T-cell–based therapy (47). CG antigens are exclusively expressed or overexpressed in tumor and germ cells. These peptide antigens are rarely presented to immune cells because of the relatively low HLA expression in testis and germ line tissues (48, 49). In order to search for these CG antigens in our MS data for class I-presented peptides, we established a customized CG antigen library by compiling 285 CG antigens from various cancer testis antigen and tumor-associated antigen DBs (Fig. 3*A* and supplemental Table S4). We identified a total of 40 CG antigen peptides derived from 14 CG antigen proteins. Of the 40 CG antigen–derived peptides, 27 are from melanoma and 13 from lung adenocarcinoma, and of these, seven were novel and, to our knowledge, have not been reported previously (Fig. 3*B* and supplemental Table S5). We selected 16 synthetic peptides for further validation and confirmed seven of these peptides from the melanoma patient–derived cell lines and one from the lung adenocarcinoma patient tumor (Fig. 3*C* and supplemental Fig. S5, *A*–*H*). We identified 15 melanocyte protein PMEL (GP100)-derived epitopes in the 3784Mel cell line derived from the tumor of a patient whose tumor-infiltrating CD8^+^ lymphocytes have been previously shown to recognize the GP100 antigen (18). Although CG antigens have been extensively studied in melanoma, we identified one novel peptide, VTPVEVHIGT, derived from sperm-associated antigen 17. In contrast, and of particular interest, we identified four novel peptides in the H1975 lung adenocarcinoma cell line. These include peptides mapping to testis-expressed protein 15 (50) and lactate dehydrogenase C (LDHC) (51) (supplemental Fig. S5*I*). Next, we verified whether the genes for these proteins were expressed at the transcript (mRNA) and protein levels in H1975 cells where the total RNA and whole-cell proteome were profiled separately (supplemental Table S6). Interestingly, expression of testis-expressed protein 15 and LDHC RNA was lower, and protein was not detected, underscoring the possibility that class I presentation can occur for genes expressed at low levels (Fig. 3*D*). We also ranked CG antigen gene and protein expression in H1975 cells and found representation of class I-associated peptides for genes with protein levels undetectable by MS but detectable at the transcript level (Fig. 3*E*). Therefore, our data support the phenomenon that immunopeptidome lacked association with gene/protein expression, which has been demonstrated previously using dynamic stable isotope labeling by/with amino acids in cell culture approach (52, 53).

### *In vivo* PTM peptides are presented by HLA class I and are potential neoantigens

**Fig. 4:**
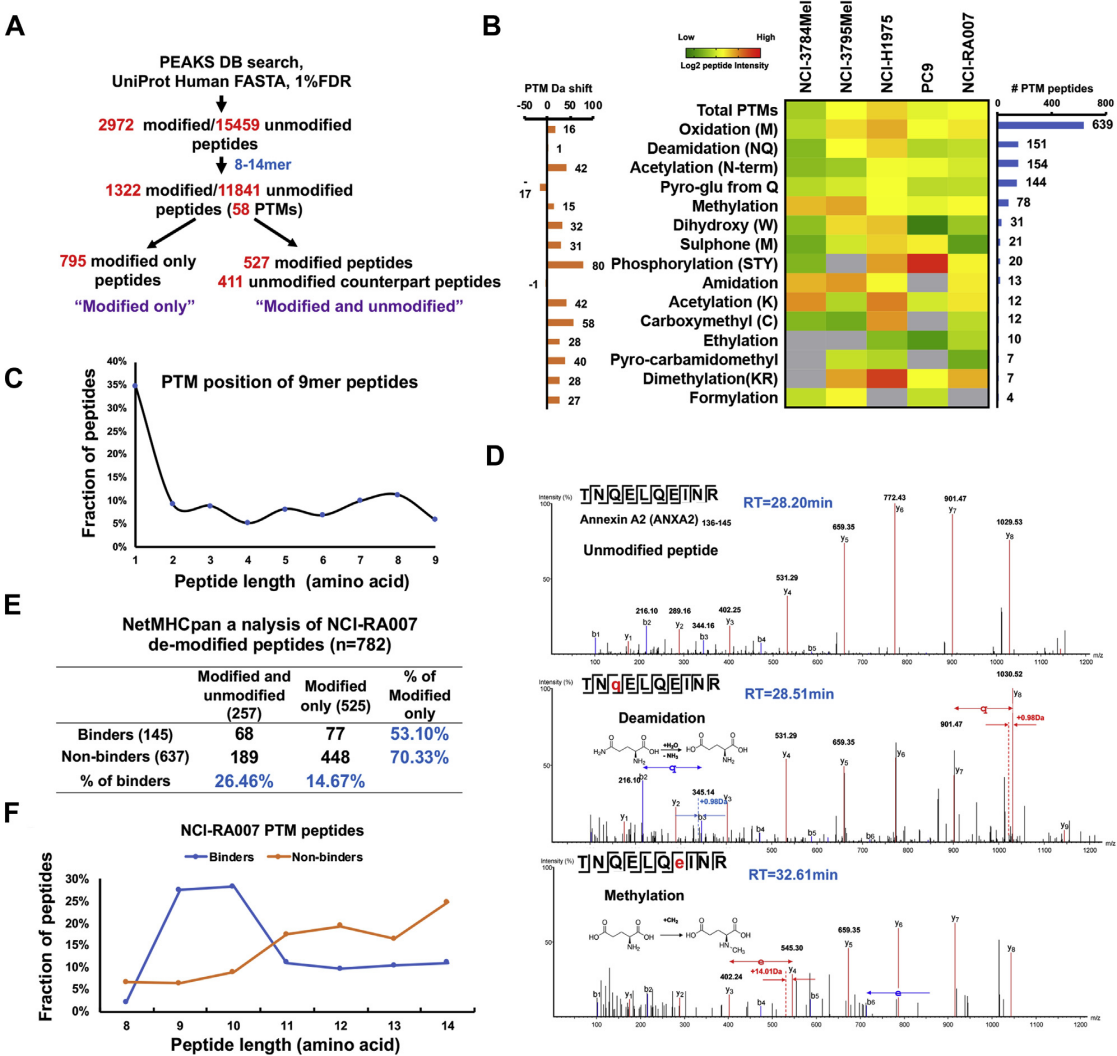
**Characterization of post-translationally modified (PTM) HLA class I-associated immunopeptides.***A*, schema of PTM immunopeptidome profiling pipeline. *B*, heat map shows the median log2 intensity of all identified class I-presented PTM peptides; *left bar graph* displays the molecular weight shift of each PTM; *right bar graph* shows the total number of peptides identified in each PTM group. *C*, PTM amino acid position distribution of the 9-mer PTM peptides. *D*, MS/MS spectra of one representative peptide with multiple PTMs. ANXA2-derived peptide TNQELQEINR, identified as unmodified (*top panel*), deamidated (*middle panel*), and methylated (*lower panel*). *E*, NetMHCpan analysis of 782 PTM peptides identified in sample NCI-RA007 where peptides with %Rank <2.0 are considered binders. The table shows the percentage of predicted “binders” in the “unmodified and modified” and “modified-only” peptides; it also shows the percentage of “modified-only” peptides in the “binders” and “nonbinders” peptides. *F*, peptide length distribution of predicted binder and nonbinder PTM peptides identified in NCI-RA007. HLA, human leukocyte antigen.

**Supplemental Figure S6:**
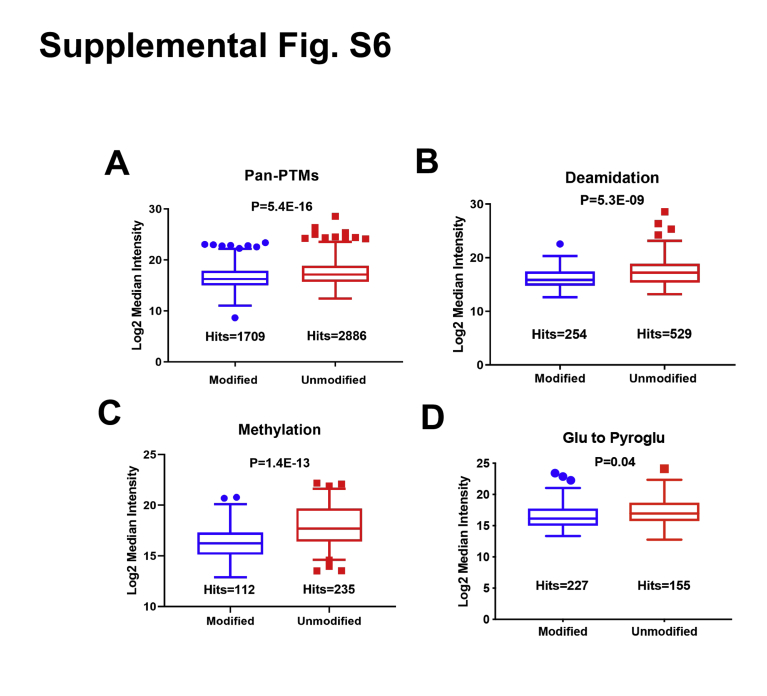


PTMs may alter the binding affinity of class I-presented peptides. Prior studies have identified PTMs in class I-presented immunopeptides (12, 14). To identify the *in vivo* PTM peptides, we used PEAKS studio with the pan-PTMs selected (over 650 variable modifications) to detect all possible PTMs. Peptide modification artifacts induced by sample preparation (*e.g.*, urea, reducing, and alkylating reagents) and electrospray ionization are the major concerns for *in vivo* PTM identification (54). Our HLA class I-presented peptidome enrichment protocol did not involve the use of urea buffer, protein reduction, and alkylation or enzyme digestion. Electrospray ionization artifacts could be excluded by examining the RT of modified peptides and their unmodified counterparts. Since the artifact modifications are only added during the ionization process, these modified peptides must be coeluted with their unmodified counterparts; yet we did not observe coeluted peptides with and without modifications. We identified 1389 modified and 11,841 unmodified 8- to 14-mer peptides. We did not detect the corresponding unmodified form for 804 of the modified peptides. These groups of peptides are defined as “modified only.” On the other hand, for 411 of the unmodified peptides, we identified 527 modified counterparts this is a group of peptides defined as “modified and unmodified,” suggesting the existence of multiple PTMs for some peptides (Fig. 4*A* and supplemental Table S7). Approximately 10% of the total peptides identified had at least one PTM, and we identified 58 different PTMs making this the largest HLA PTM immunopeptidome identified to date. The heat map shows the median intensity of each PTM in each sample whereby methionine oxidation (639 peptides), deamidation (151 peptides), acetylation (144 peptides), and methylation (78 peptides) were the most abundant modifications seen (Fig. 4*B*) in agreement with previous reports (12, 13). Interestingly, among the 9-mer peptides identified, the N-terminal amino acid was most commonly modified; the first amino acid was modified in 102/293 9-mer PTM peptides (Fig. 4*C*). PTM HLA peptides were previously shown to be more abundant than their unmodified counterparts (12). However, in our dataset, pan-PTM peptides were significantly less abundant (as measured by peptide intensity) than unmodified peptides (*p* = 5.4E-16) (supplemental Fig. S6*A*). Similarly, MS intensity was lower for deamidated and methylated peptides than unmodified peptides (supplemental Fig. S6, *B* and *C*). However, the median intensity of glutamate- to pyroglutamate (pyroGlu)-modified peptides was similar to that of their unmodified counterparts (supplemental Fig. S6*D*). The pyroGlu modification occurred on glutamate at N-terminal position 1, which does not significantly affect the peptide conformation and binding affinity (55).

To validate the data quality and verify that the modifications were generated *in vivo* and not experimental artifacts, we selectively examined the tandem mass spectra of the modified peptides. A deamidated and a methylated form of peptide TNQELQEINR, derived from annexin A2, had an RT of 28.51 and 32.61 min, respectively, whereas the unmodified peptide had an RT of 28.20 min (Fig. 4*D*). We manually verified the RT of methionine-oxidized peptides and their unaffected counterparts and determined that they did not coelute during LC, suggesting that these PTM peptides likely were generated *in vivo* and were not ionization artifacts. Next, we determined whether the PTMs may alter the binding affinity to HLA class I. There are no HLA-binding prediction algorithms commercially available that accounts for PTM peptides. Using the unmodified forms of the peptides identified for NetMHCpan analysis of tumor tissue from NCI-RA007, 637 of 782 PTM peptides were considered to be nonbinders (%Rank >2.0), suggesting that specific modifications of these peptides may have been critical for HLA binding. Notably, the percentage of predicted binders for peptides with both modified and unmodified counterparts was nearly twice (26.46%) that of solely modified (14.67%). Also, the percentage of modified-only peptides among predicted binders (53.1%) was much lower than for nonbinders (70.33%) (Fig. 4*E*). We plotted peptide length distribution of the binders and nonbinders and found that they had a similar pattern although more nonbinders were longer peptides (>10-mer) (Fig. 4*F*). This further suggests that PTM peptides may have nonconventional length distribution for HLA class I binding; importantly, the unmodified forms of these peptides are more likely to be predicted as “nonbinders.” Collectively, our findings suggest that PTMs may play a crucial role for generating a subset of HLA class I-binding peptides with unique binding motifs for antigen presentation.

### *De novo* sequencing provides reliable immunopeptide identification

**Fig. 5:**
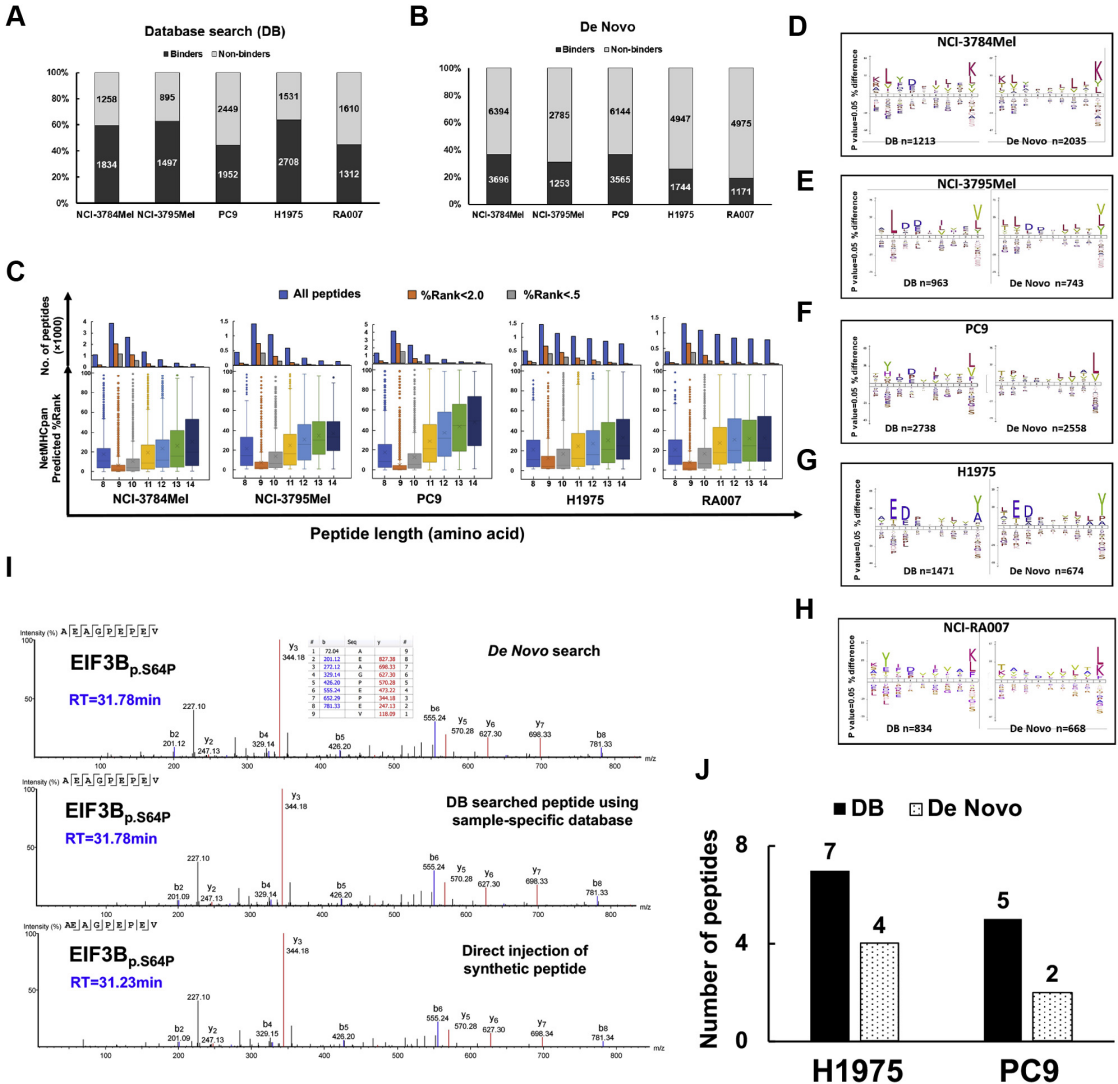
***De novo* search of MS data to identify class I-associated immunopeptides.***A* and *B*, fraction of class I-associated peptides predicted to be HLA binders (NetMHCpan %Rank <2.0) or nonbinders (%Rank >2.0) by *A*, database (DB) search and *B, de novo* sequencing. *C*, NetMHCPan predicted %Rank distribution of total number of *de novo* sequencing–searched 8- to 14-mer peptides; HLA binders (%Rank <2.0) and strong binders (%Rank <0.5) (*upper panel*). Distribution of HLA-binding affinity (%Rank) of *de novo* sequencing–searched 8- to 14-mer peptides (*lower panel*). 9-mer peptides have the lowest predicted %Rank, suggesting the strongest binding. *D*–*H*, comparison of 9-mer peptide-binding motifs (binders only, NetMHCPan %Rank <2.0) identified by DB search (*left*) *versus de novo* search (*right*) in *D*, NCI-3784Mel, *E*, NCI-3795Mel, *F*, PC9, *G*, H1975, and *H*, NCI-RA007. *I*, matched MS2 spectra and RT of one representative endogenous variant peptide, EIF3B_p.S64P_ (AEAGPE*P*EV), identified by *de novo* search (*upper panel*), proteogenomic DB search (*middle panel*), and direct injection of its synthetic peptide (*lower panel*). *J*, bar charts show that variant peptides identified by proteogenomic analysis were also retrieved in the *de novo*–only search. HLA, human leukocyte antigen; RT, retention time.

**Supplemental Figure S7:**
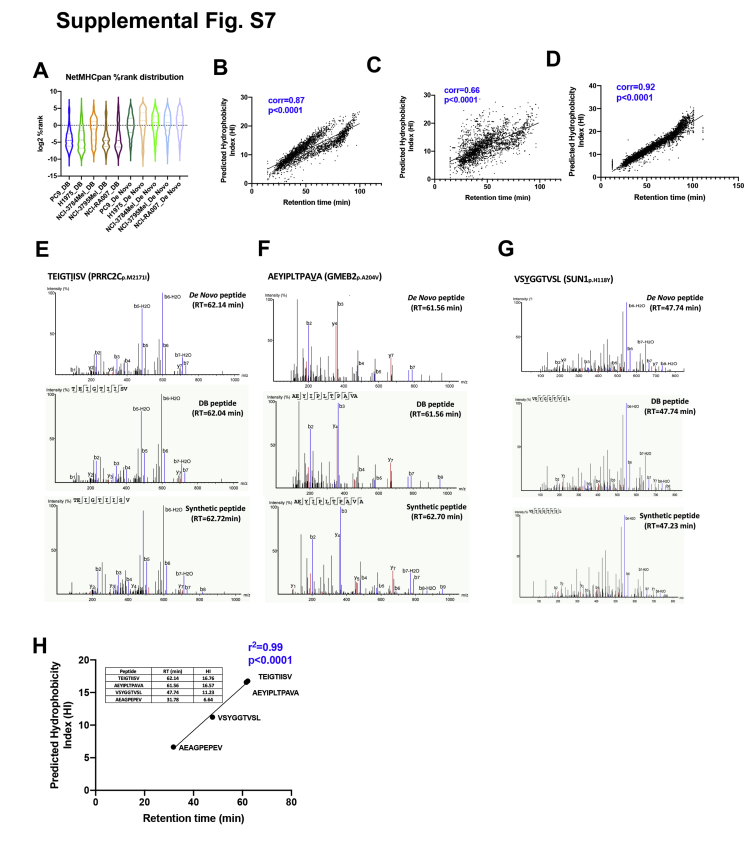
7

*De novo* search of MS spectra from large-scale MS data has been employed by various algorithms, including PEAKS studio. The impressive prediction accuracy of this approach has been extensively reported (24, 56, 57). We searched our entire class I-presented immunopeptidome MS data using the PEAKS studio *de novo* search algorithm. First, to evaluate the data quality of the peptides identified by *de novo* sequencing, we manually inspected the MS2 spectra. We then employed the NetMHCpan prediction to evaluate the binding capacity of our *de novo*–only peptides (not including DB-searched peptides). We determined that while an average of ∼55% of the DB-searched peptides were predicted to be specific HLA allele binders for their corresponding cell line/tissue, an average of ∼33% of *de novo* peptides were predicted to be a strong binder of at least one HLA allele in the respective sample (Fig. 5, *A* and *B*), the distribution of %Rank of DB and *de novo* search peptides in each sample is shown in supplemental Fig. S7*A*. The predicted HI and RT of 9-mer peptides identified by DB and *de novo* search showed significant correlations while, as predicted, the HI and RT of the tryptic peptides showed even higher correlation (supplemental Fig. S7, *B* and *D*). We employed a similar approach to evaluate the HLA-binding affinity of 8- to 14-mer peptides identified by DB search (Fig. 1*D*) by predicting the binding affinity and assigning each peptide to its highest predicted HLA allele and showing the distribution of 8- to 14-mer peptides according to their lowest %Rank. We observed the same trend with respect to binding score distribution of 8- to 14-mer peptides when comparing *de novo* to DB-identified peptides. The 9-mer peptides have the lowest binding scores and hence highest binding affinity to the best predicted HLA-binding allele (Fig. 5*C*). To further confirm the validity of the peptide identities from the *de novo* sequencing analysis, we compared the predicted DB and *de novo* 9-mer HLA-binding peptide motifs. Interestingly, the peptide motifs were very similar between the two groups, reinforcing the validity of the *de novo*–identified peptides generated from all five samples (Fig. 5, *D*–*H*). We next aligned the *de novo* and DB search spectra for four representative endogenous variant peptides identified in H1975. The b and y ions of the spectra from the endogenous variant EIF3B_p.S64P_ peptide (AEAGPEPEV) (Fig. 5*I*) as well as three additional variant peptides (supplemental Fig. S7, *E*–*G*) perfectly aligned with the spectra from corresponding synthetic peptides. Furthermore, the predicted HI and RT of these four *de novo* peptides identified in H1975 were well correlated with *r*^2^ = 0.99 (supplemental Fig. S7*H*). We further validated our *de novo* sequencing pipeline-identified variant peptides from the lung adenocarcinoma cell lines by comparison to those identified using cell line–specific DB search. Indeed, we found four of seven and two of five mutant peptides in H1975 and PC9 cells with relatively high confident ALC%, respectively (Fig. 5*J*). In summary, we present strong evidence that this is a robust and reliable *de novo* sequencing pipeline for MS identification of the immunopeptidome.

### Identification of lnc-RNA–derived peptides using *de novo* sequencing

**Fig. 6:**
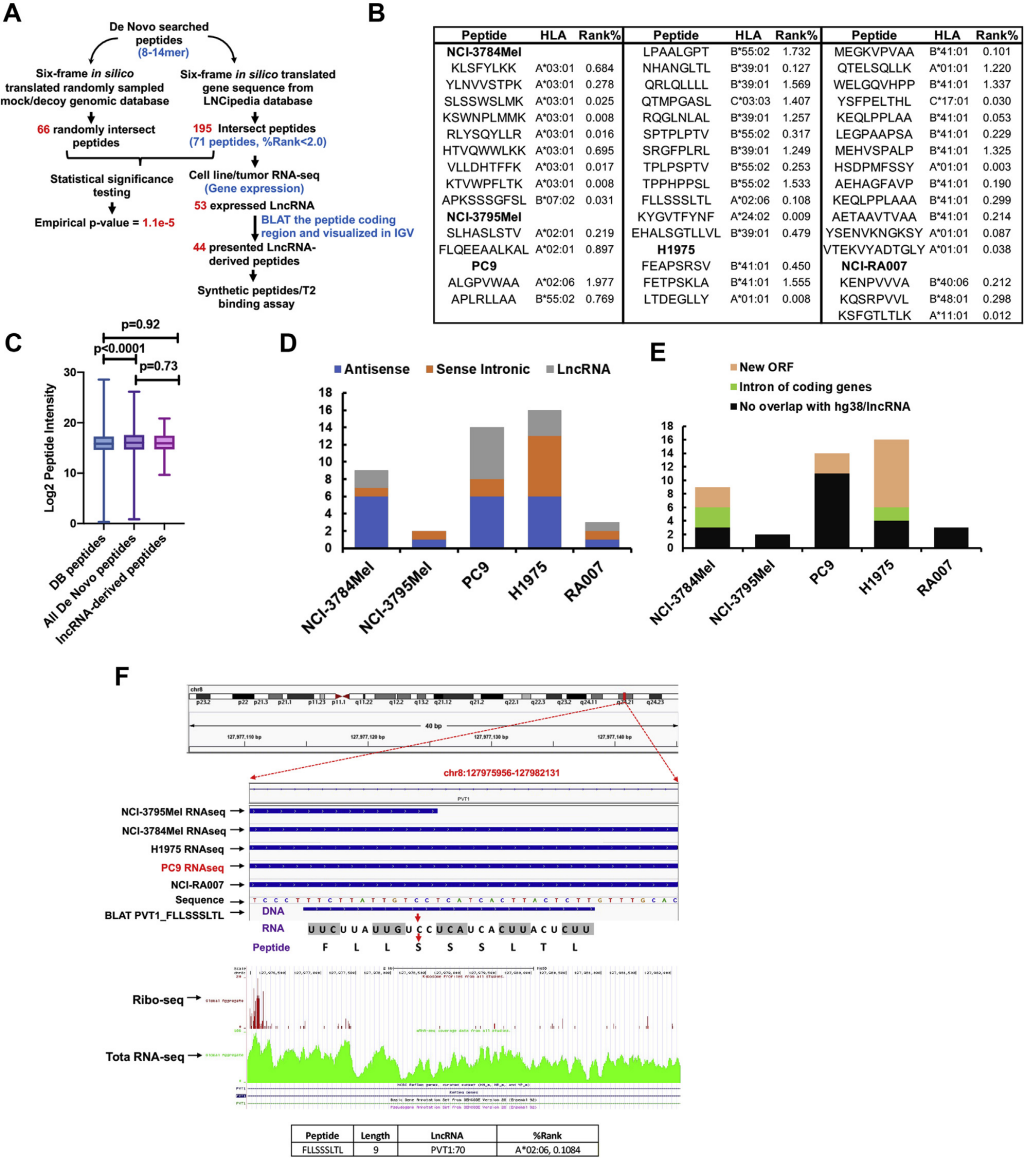
**Identification of lncRNA-derived peptides by proteogenomic and *de novo* sequencing analyses.***A*, schema showing the workflow used to identify lncRNA-derived peptides enriched from cancer cells and tumors. The *de novo*–only sequencing-searched class I-presented peptide pool was queried against a database (DB) generated using six-frame translated lncRNAs compiled in LNCipedia DB (*right workflow*). The statistical significance of our algorithm was determined by potential matching of the *de novo*–searched peptides against a “mock” DB created by the randomly picked gene blocks (~50,000 transcripts) from hg38, which resulted in an empirical *p* value <1.0e^−5^ (*left workflow*). *B*, 44 lncRNA-derived peptides identified using our algorithm with their predicted HLA alleles and binding affinity. *C*, comparison of log2 peptide intensities of DB searched, *de novo* searched, and lncRNA-derived peptides. *D*, the classification of source lncRNAs for the identified lncRNA-derived peptides into antisense, sense intronic, and classic lncRNAs. *E*, lncRNA-derived peptides that match new ORF, introns of coding genes, and noncoding region. *F*, *top panel* displays a snapshot of IGV showing lncRNA PVT1-derived peptide FLLSSSLTL, identified in PC9, with chromosomal location alignment of RNA-Seq of all five samples and peptide BLAT; the *middle panel* shows the ribo-seq searching results from GWIPS; the *lower panel* shows the predicted binding affinity of this peptide to HLA-A∗02:06 that is expressed only in PC9 cells. BLAT, BLAST-like Alignment Tool; GWIPS, Genome-Wide Information on Protein Synthesis; HLA, human leukocyte antigen; IGV, Integrative Genomics Viewer; lncRNA, long noncoding RNA.

Noncoding regions in the genome are the most unexplored; yet they are rich sources of neoantigens. Previous studies have profiled the noncoding immunopeptidome using the traditional proteogenomic approach of searching the MS raw files against sample-specific library generated from RNA-Seq data (58, 59), which always resulted in extremely massive customized noncoding sequence libraries because 99% of the human genome is noncoding (60), a majority of which are unannotated. We developed a pipeline to profile potential lncRNA-derived peptides by taking advantage of deep *de novo* analysis of MS data without using a predefined DB and then matching the MS-identified peptides with hypothetical peptides generated by six-frame translation of all lncRNAs from an available lncRNA DB, LNCipedia, containing 49,372 lncRNAs from the high-confidence genome assembly (33). The 8- to 14-mer *de novo* peptides were queried against all six potential reading frames of the translated LNCipedia-derived protein DB. We also confirmed the transcript expression of the lncRNAs coding the identified peptides in RNA-Seq gene expression data from the patient-derived cell lines. IGV was used to visualize peptide coding regions of these lncRNAs. We finally validated the endogenous peptides derived from the lncRNAs with synthetic peptide-based spectra matching and T2 cell–based HLA stability assay for binding to specific HLA alleles (Fig. 6*A* and supplemental Fig. S8*A*). A total of 195 distinct *de novo* sequencing–identified peptides matched to the six-frame translated lncRNAs in the LNCipedia DB, of which, 71 were predicted to be binders (%Rank <2.0) for at least one HLA allele in their corresponding cell line/tumor. We further analyzed the RNA-Seq data and found that the source RNAs of 53 peptides were transcribed. The feature counts of these transcribed lncRNAs were displayed in a heat map (supplemental Fig. S8*B*). We then confirmed 44 peptides had their specific coding regions transcribed and did not overlap with any protein-coding region using the BLAT in IGV (Fig. 6*B* and supplemental Table S8). Two representative examples of the data visualization using IGV for the peptides identified from lnc-JAM3-3:12 and lnc-LRP5-1:11 are shown (supplemental Fig. S8, *C* and *D*). Notably, we did not observe any peptide presented in more than one sample, implying that lncRNA-derived peptides may be tumor cell line specific and patient specific. To assess the significance of our findings, we generated a mock lncRNA pool from randomly sampled ∼50,000 genomic sequences. We rejected the null hypothesis that our identified lncRNA-derived peptides randomly matched to a six-frame–translated LNCipedia-derived DB, as we obtained a significant empirical *p* value (1.1e^−5^) upon comparing to the random matches against the mock lncRNA pool-derived six-frame translated DB.

Next, we asked whether lncRNA-derived class I-presented peptides had low abundance. We found that lncRNA-derived peptides were equally presented on class I as all other DB and *de novo* sequencing–derived peptides (Fig. 6*C*). Based on LNCipedia classification, the source lncRNAs matched to 20 antisense genes, 12 sense intronic genes, and 12 lncRNA genes (Fig. 6*D*). Furthermore, BLAT analysis revealed that 23 lncRNA-coding regions had no overlap with any coding region on hg38; five lnRNAs matched to the introns of coding genes, and 16 matched to novel ORFs because of the frameshift that intersected with exons of known protein-coding genes, but with different start codons (Fig. 6*E*). Frameshifted new ORFs have been suggested to be a rich source of neoantigens (61).

**Fig. 7:**
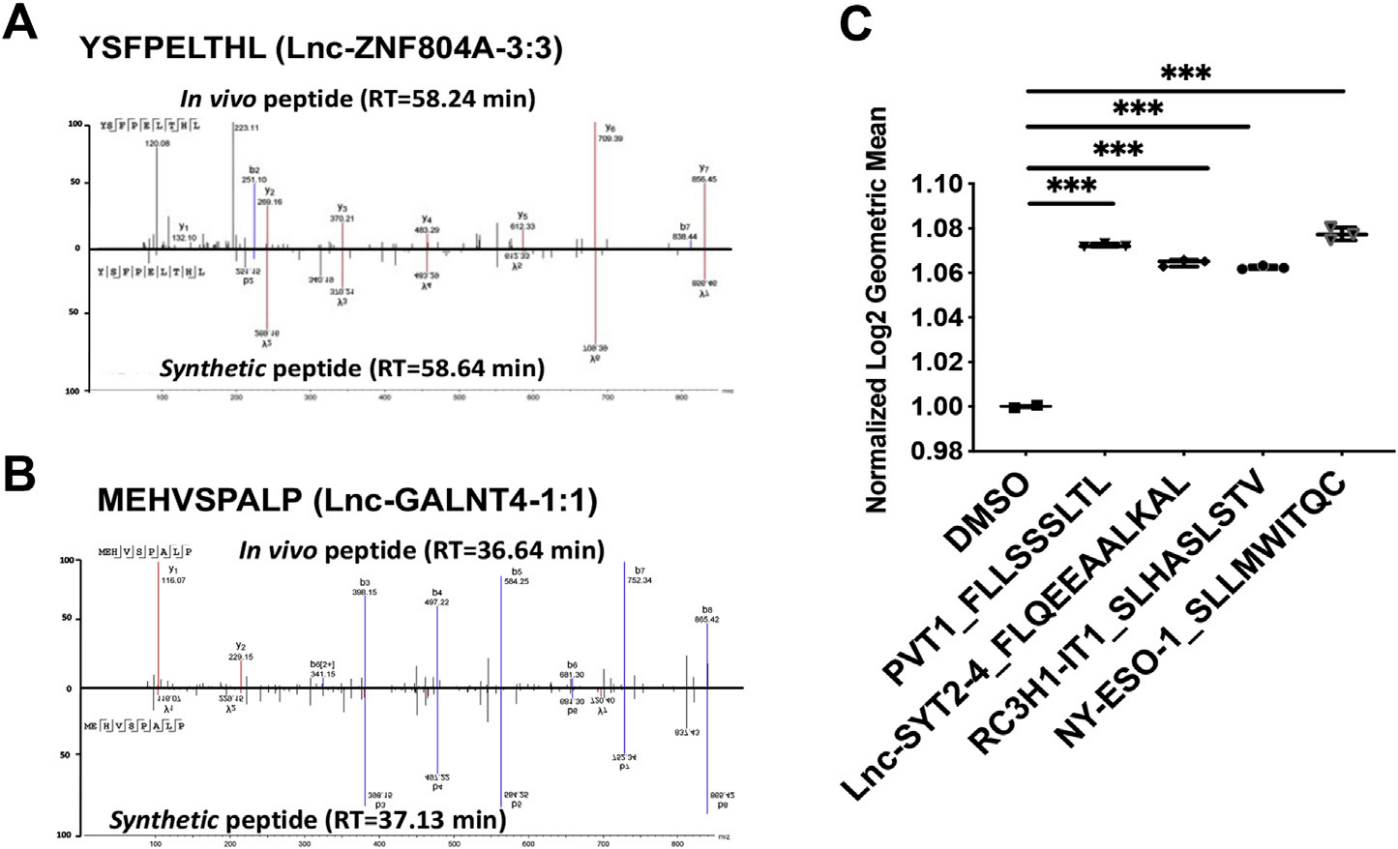
**Validation of lncRNA-derived peptides using synthetic peptides and T2 binding assay.***A* and *B*, MS2 spectra matching of two lncRNA-derived peptides and their synthetic counterparts, YSFPELTHL and MEHVSPALP. *C*, T2 cell-based HLA stability assay showing the three lncRNA peptides predicted to be HLA-A∗02 binders, FLLSSSLTL, QEEAALKAL, and SLHASLSTV, and the NY-ESO-1-derived positive control peptide binding and stabilizing the HLA allele. HLA, human leukocyte antigen; lncRNA, long noncoding RNA.

It remains unclear whether lncRNA can be translated to protein products (*e.g.*, full-length/truncated proteins, peptides), and importantly, presented to cell surface by HLA class I. For in-depth illustration of our computational strategy, we analyzed further the lncRNA oncogene, *PVT1*-derived 9-mer peptide, FLLSSSLTL, identified in PC9 cells (Fig. 6*F*). RNA-Seq BAM files were converted to BED files for all five samples. The DNA coding sequence of the identified peptide, FLLSSSLTL, was retrieved from the *PVT1* nucleotide sequence in LNCipedia. The BLAT results of this 27-base pair sequence (*i.e.*, TTC … CTT) were *in silico* transcribed to RNA sequence (*i.e.*, UUC … CUU) and further translated to peptide sequence FLLSSSLTL. Interestingly, we found that the source lncRNA was transcribed in all five samples, but a truncated version was transcribed in NCI-3795Mel. However, the peptide was only identified in PC9 cells, suggesting that translation of lncRNA-derived peptides may be cell specific and context specific, or the presentation of lnc-RNA–derived peptides by class I is cell line specific or tumor specific. We reasoned that the specificity to PC9 cells could be a result of the lack of specific HLA allele to present this A∗02 restricted peptide (%Rank = 0.11). Only PC9 and NCI-3795Mel express HLA-A∗02, and NCI-3795Mel expresses a truncated version of FLLSSSLTL; this may explain why we only observed this peptide presented by class I in PC9 cells. We further confirmed that the ORF containing this peptide coding sequence has been reported in the ribosome profiling data based on deep sequencing of ribosome protected mRNA fragments that can be visualized using GWIPS-viz (38). We further confirmed our MS identification of these lncRNA-derived peptides that are presented by HLA class I. We used synthetic peptides and performed LC-MS/MS to compare the MS2 spectra of synthetic peptides with those of the endogenous peptides (Fig. 7, *A* and *B*). Finally, we used the T2 cell–based HLA stability assay to confirm that three lncRNA-derived peptides from the lncRNAs, PVT1, Lnc-SYT2-4, and RC3H1-It1, were truly HLA-A∗02 binders (Fig. 7*C*). Taken together, we report a novel MS-based class I-associated peptidome profiling platform for identification of lncRNA-derived peptides that are presented by HLA class I.

## Discussion

Neoantigens or cancer-associated antigens are attractive immunotherapeutic targets because they specifically engage T-cell receptors in T cells that promote an immune response against the tumor tissue while sparing nearby healthy tissues. Emerging evidence obtained from breast cancer (62), bladder cancer (63), melanoma (64), and lung cancer (65) studies suggests that cancer neoantigens and cancer-associated antigens may be ideal targets for ACT and therapeutic cancer vaccines. MS-based peptide sequencing technology provides direct experimental evidence for a large number of HLA-presented peptides. As such, this approach has become a robust and quick method of neoantigen discovery (10, 13, 66). Nevertheless, a majority of studies have focused on tumor types with high TMB, such as melanoma (14, 67). EGFR-mutant lung cancer, with low TMB, and tumors with loss of neoantigen expression while on immunotherapy are relatively resistant to immune checkpoint therapy (68, 69, 70). In this study, it was our intent to leverage discovery proteomics and informatics to identify HLA class I-presented peptide antigens, including common driver oncogenes, variant peptides, CG antigen peptides, PTM peptides, and lncRNA-translated peptides for immunotherapy in EGFR-mutant lung adenocarcinoma, a tumor type that is historically less responsive to immune checkpoint inhibitor therapy for variety of reasons, including low TMB. Moreover, we utilized spectra matching between *in vivo* peptides and synthetic peptides as well as HLA-binding assays to further validate our identification of class I-presented peptides. We acknowledge that this study contains relatively small sample size; nonetheless, it readily represented two cancer types, melanoma and lung cancer, and two types of specimen, cancer cell line and tumor tissue. To our knowledge, such a large-scale study has not been performed in EGFR-mutant lung cancer which does not respond to traditional checkpoint inhibitor therapy. Some of the identified peptides derived from tumor-specific antigens or tumor-associated antigens may be further validated and be candidates for developing precision immunotherapy.

To confirm the validity of the class I immunopeptidome identified in this study, we first verified the quality of our DB-searched HLA class I-presented peptides by confirming that a majority of the peptides have high binding scores against their cognate HLA alleles expressed in the source tumors and cell lines. This is consistent with previous large-scale monoallelic HLA class I epitope profiling studies (13, 71). The NetMHCpan-predicted binding scores of the identified peptides favor 9- and 10-mer peptides that are of the optimal length for class I presentation (Fig. 1*D*). Combining SNVs, INDELs, and fusion variants, our customized search algorithm was able to identify five COSMIC-reported mutation-containing peptides, of which, FHAQNIHQTF, derived from somatic mutation CIP2A_p.R229Q_ has been associated with several solid cancers, including lung adenocarcinoma, colon cancer, and pancreatic cancer (Fig. 2*C*). No significant correlation was observed between total mutation burden and HLA presentation in the low and high TMB cancers. Similar identification of modestly large HLA peptidome that was reported in a low TMB cancer, such as renal cell carcinoma, supports our findings (72). One possible interpretation is that total number of identified class I-presented immunopeptides more directly relates to the purified amounts of HLA proteins and its allotype diversity, and that is similar between our chosen patient-derived cell lines (Table 1 and supplemental Fig. S1*D*). Some of the mutated peptides may not be presented by HLA class I; rather, specific variant peptides are still presented in low TMB cancers, and identification of those by direct MS will be beneficial for designing precision immunotherapies. We acknowledge that class I- and class II-presented peptides harboring the truncal mutations in common oncogenes, such as *EGFR* and *KRAS*, may be the most attractive targets for ACT (8, 65). We have reported the identification of somatic mutated peptides by MS from the proteome of patients with lung cancer, including a novel somatic mutated CDK12_p.G879V_ peptide using similar methodology (19). Although we identified 19 peptide epitopes derived from common oncogenes, none of them contained somatic mutations (supplemental Fig. S3*F*). Possible explanation of the identification of relatively few mutant peptides from known oncogene mutations include the absence of the cognate class I allele and limitations of the data-dependent acquisition methodology for MS-based sequencing of peptides when the mutated peptides are just a minor fraction of the total wildtype peptides presented by HLA class I. However, interestingly, the median MS intensity of the few neoantigens we identified is very similar to that of wildtype peptides (*p* value > 0.05), indicating that select neoantigens are robustly presented by class I.

We have generated the most comprehensive CG antigen DB to date by leveraging the human proteome atlas and multiple peer-reviewed CG antigen DBs. This is a valuable resource that can be used to query CG antigens from other large cohort immunopeptidome studies. For instance, we identified LDHC (supplemental Fig. S5*I*) which, prior to this study, was almost exclusively observed in testis, and an association with lung adenocarcinoma had only been suggested (51). Given that CG antigens have been extensively investigated in melanoma (73, 74), we have now described the CG antigen landscape in EGFR-mutant lung cancer, unveiling novel class I-presented peptides reported in our study. As previously reported, our results also showed that CG antigen–derived peptide levels are not significantly correlated with mRNA and source protein expression (40, 75, 76).

One of the advantages of a PEAKS DB search is its pan-PTM search engine, which unveils all PTMs in one search, requiring no prior knowledge of the potential types of modifications expected. PeaksPTM uses the sequence-tag approach to identify PTMs without generating an extremely large sequence library containing variable modifications (77, 78). This allows identification of many PTMs in one search. To our knowledge, this study provides the deepest coverage of the PTM HLA class I immunopeptidome, to date. Compared with previous profiling of PTM immunopeptides (12, 14), we, for the first time, systematically quantified the largest number of endogenous PTMs of class I-presented peptides in EGFR-mutant lung adenocarcinoma. We demonstrated that unmodified peptides were significantly more abundant than their deamidated or methylated counterparts. However, this does not apply to the conversion of glutamate to pyroGlu, where a modification could lead to protein misfolding (55). Generally, low peptide abundance or presentation in antigen-presenting cells may hinder T-cell recognition. It is possible that more abundant peptides are more likely represented as T-cell epitopes. Although phosphorylation is the most dominant PTM known to modulate cellular function, we identified only a minor fraction of class I-presented peptides phosphorylated; this could be due to the relatively large phosphate group, which may not easily fit into the HLA-binding groove. The binding of only around two thousand unique phosphopeptides to 72 HLA alleles has been reported (79). The transient reversible PTM phosphorylation generates less-stable peptides that are less likely to be good targets for HLA class I presentation. This is in contrast to irreversible PTMs such as deamidation, which are more likely to generate neoepitopes. We identified 20 deamidated peptides with a NX(S/T) motif, which supports the potential mechanism of asparagine deamidation that deamidated immunopeptides are derived from deglycosylation (80). We discovered that a single peptide could have multiple PTMs concurrently (Fig. 4*D*). Position 1 was the most frequently modified (Fig. 4*C*), which implies that small modifications on a nonanchor position may not dramatically affect binding affinity. We compared the predicted binding score of the modified-only peptides with the peptides that were identified as both modified and unmodified versions. The modified-only peptides have lower binding scores using NetMHCpan prediction (Fig. 4, *E* and *F*), indicating these peptides may possess a unique HLA-binding domain structure that is modification dependent and cannot be analyzed by available prediction algorithms. However, we acknowledge that a limited fraction of PTM peptides may occur spontaneously *in vitro*, although the sample preparation was conducted at 4 °C. For example, deamidated peptides with an NG motif have been reported as *in vitro* artifacts (81). Taken together, our results demonstrate that the PTM peptides that are not predicted by any bioinformatics algorithm for HLA binding can be identified using MS and these peptides may be a rich source of potential neoepitopes.

Deep learning–based *de novo* search algorithm embedded in PEAKS provided high accuracy to detect peptides that normally are missed in a DB search. Our pipeline evaluates in-depth the *de novo*–only peptides in class I immunopeptidome context. The validity of this approach for HLA peptidome profiling and variant peptide identification is underscored by several findings in this study. Although the identified *de novo*–only peptides may constitute many spuriously identified peptides, which are not predicted to be binding to any HLA allele in respective samples, we believe that PEAKS *de novo* search unveiled reasonable fraction of true HLA class I-presented peptides considering no decoy DB is available to control the FDR (Fig. 5, *A* and *B*). We acknowledge that the correlation of SSRCalc-predicted HI and RT of tryptic peptides is higher than that of immunopeptides (supplemental Fig. S7, *B*–*D*). This indicated potential higher FDR in *de novo* search for HLA peptides. The lower correlation could also be a result of inaccurate prediction of HI by SSRCalc for nontryptic peptides, such as the HLA class I-presented 9-mer immunopeptides. SSRCalc was originally designed for tryptic peptides, which end with either lysine or arginine and always has been reduced and alkylated before LC–MS/MS analysis (82). Also, nontryptic HLA peptides usually have shorter length (8- to 12-mer) than tryptic peptides (8- to 25-mer). PEKAS overcomes this high FDR problem by using ALC scoring for peptide identification (83). The binding motif of the 9-mer *de novo*–only peptides was very similar to the DB-searched peptides (Fig. 5, *D*–*H*). MS/MS sequencing spectra of select variant peptides in H1975 identified from the *de novo* search and proteogenomic DB search were a perfect match to the corresponding synthetic peptides (Fig. 5*I* and supplemental Fig. S7, *E*–*G*). Half of the *de novo*–only searched variant peptides from H1975 and PC9 identified by the proteogenomic pipeline were also identified using our customized human proteome DB (Fig. 5*J*). We further validated the variant peptides identified by *de novo* search algorithm by matching the predicted HI and RT of the select four variant peptides from H1975 that were also validated by spectra matching with synthetic peptides. Further confirmation and validation of the variant and PTM peptides identified by *de novo* sequencing by the PEAKS search algorithm can be obtained by spiking synthetic heavy-labeled peptides and demonstrating coelution of endogenously identified peptides with the heavy-labeled synthetic peptides. Overall, our results suggest that *de novo* search by PEAKS identifies high-quality class I-presented peptides and is a powerful tool to identify noncanonical peptides/proteins for proteogenomic profiling.

Noncoding RNA is recognized as a rich resource for neoantigens (84), and MS-based proteogenomic platforms have been implemented to discover noncanonical peptides (15, 57). Laumont *et al.* (58) suggested that noncoding regions are the main source of neoantigens. In contrast to previous studies where MS spectra were mapped *in silico* to all potential reading frames of FASTA-derived RNA-Seq data, we leveraged deep learning methodology of *de novo* sequencing of peptides in PEAKS studio to query against the largest annotated lncRNA DB. Our approach extends the previous studies in many ways. One of the challenges in proteogenomic field is that the addition of genomic sequencing *in silico*–translated DB can result in a higher chance of false identifications (85). Since LNCipedia DB consolidates many datasets from a variety of sources and contains bidirectional lncRNAs in which transcription can be initiated from both ends (33), we constructed a six-frame–translated DB derived from the lncRNA DB and interrogated potentially matched *de novo*–only searched peptides from the PEAKS search engine. As has been reviewed before, false-positive matched peptides increase upon interrogation of such large DBs (86). However, the highly significant *p* values obtained by our empirical *p* value calculation, similar to a permutation test, on the mock lncRNA DB, performed exactly the same way considering all six reading frames for a potential peptide match reduces the possibility that there are significant false positives in the identified lncRNA-derived immunopeptides. In addition, we visualized and manually verified that the lncRNA-derived peptide coding regions do not overlap with any protein-coding regions, introns of known protein-coding genes, or could result from a frameshift. We also selectively validated the presence of the source lncRNAs in the ribosome profiling data searching the GWIPS DB, suggesting that these lncRNAs are indeed translated on the ribosome machinery. To further ensure that the identified lncRNA-derived peptides were indeed not a part of a known expressed protein, we manually checked and visualized that the source lncRNA of each of these novel peptides was indeed present in the total RNA-Seq data from the same cell line and tumor and did not overlap with any coding region or was not because of a frameshift. Synthetic peptide validation and T2 cell HLA-binding assays further confirmed that the identified lncRNA-derived peptides were indeed presented and had high affinity to the HLA proteins. Taken together, our results show that lncRNA-derived immunopeptides are presented by HLA class I in tumors with both high and low TMB. The pipeline we developed in this study could be readily applied to any type of cancer to identify lncRNA-derived peptides presented by HLA class I.

Taken together, we report the largest characterization of potential cancer-associated class I immunopeptidome in EGFR-mutant lung adenocarcinoma to date. The combination of genomics, proteomics, and informatics allows us to develop this in-depth immunopeptidome-based cancer epitope profiling pipeline. Our results suggest that low TMB tumors possess as many potential immunotherapy-targetable epitopes as high TMB tumors, the identification of which needs in-depth analysis of MS data. We provide a valuable resource of EGFR-mutant lung cancer–specific neoepitopes as well as tumor-associated immunopeptides for possible design of precision immunotherapy and cancer vaccines.

## Data availability

The raw MS-based sequencing files of HLA class I immunopeptidome and whole-cell proteome and patient-/cell line–specific FASTA files have been deposited to ProteomeXchange *via* PRIDE. Data can be retrieved with the identifier PXD022949. All annotated MS/MS spectra of MaxQuant searched results can be found on MS-viewer (https://msviewer.ucsf.edu/prospector/cgi-bin/msform.cgi?form=msviewer) using search key “yqns2kpjew.”

The genomic and transcriptomic data have been published by our group, and the original files can be accessed *via* our previous publications (16, 18).

## Supplemental data

This article contains supplemental data.

## Conflict of interest

U. G. has a clinical trial agreement with AstraZeneca and had received research funding from AstraZeneca, Aurigene, and Esanex. U. G. is currently an employee of Bristol-Myers Squibb. The other authors declare no competing interests.

## Figures and Tables

**Supplemental Figure S8:**